# Characteristics of calcified nodule attributable to culprit lesion in acute coronary syndrome: A systematic review and meta-analysis

**DOI:** 10.1016/j.isci.2024.110351

**Published:** 2024-06-22

**Authors:** Roy Bagus Kurniawan, Pandit Bagus Tri Saputra, Alyaa Ulaa Dhiya Ul Haq, Dinda Dwi Purwati, Citrawati Dyah Kencono Wungu, Hendri Susilo, Mochamad Yusuf Alsagaff, Indah Mohd Amin, Yudi Her Oktaviono

**Affiliations:** 1 Faculty of Medicine, Universitas Airlangga, Surabaya, Indonesia; 2 Department of Cardiology and Vascular Medicine, Faculty of Medicine, Universitas Airlangga, Surabaya, Indonesia – Dr. Soetomo General Academic Hospital, Surabaya, Indonesia; 3 Department of Physiology and Medical Biochemistry, Faculty of Medicine, Universitas Airlangga, Surabaya, Indonesia; 4 Center of Preclinical Science Studies, Faculty of Dentistry, Universiti Teknologi MARA, Selangor Darul Ehsan, Malaysia; 5 Institute of Tropical Disease, Universitas Airlangga, Surabaya, Indonesia

**Keywords:** Cardiovascular medicine, Health sciences, Medical specialty, Medicine

## Abstract

The presence of calcified nodule (CN) is a significant characteristic of atherothrombosis in acute coronary syndrome (ACS). However, its characteristics continue to be understudied. This review aimed to further investigate these characteristics. This study found that CN was a distinctive feature of an atheromatous plaque, representing 6.3% of ACS. CN was more common in NSTE-ACS than in STEMI patients (9.4% vs. 6.6%). CN was also chiefly observed in the left anterior descendant artery (48%), followed by the right coronary (40.4%) and left circumflex (14.5%) arteries. Higher prevalence of hypertension (78.8%), diabetes mellitus (50.8%), multivessel disease (71.7%), and kidney disease (26.43%) were noted in CN compared to non-CN patients. CN-associated ACS also 6-fold increased the risk of target lesion revascularization compared to those without CN.

## Highlights

• CN prevalence in ACS is 6.3% and is more common in NSTE-ACS than STEMI
• DM, hypertension, MVD, and CKD are higher in individuals with CN
• CN is commonly observed in LAD and RCA than in LCx
• CN increases six-fold the risk of having target lesion revascularization

## Graphical abstract

**Figure:**
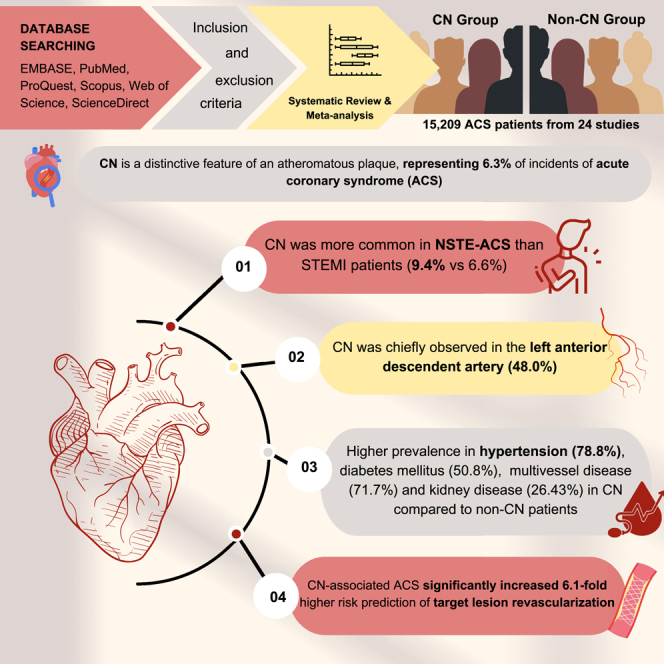

## Introduction

Acute coronary syndrome (ACS) is one of the leading causes of morbidity and mortality in the world.^1^ It is estimated that 15.5 million people over 20 years old in America have coronary artery disease and one of them dies every 42 seconds.^2^ An abrupt thrombotic complication of atherosclerotic plaque, with or without concomitant vasospasm, is the fundamental etiology of ACS.^3^ Histopathology examination demonstrated that there are three major morphological features in atherothrombosis events: plaque rupture (∼60%), plaque erosion (∼30%), and calcified nodule (<10%).^4^

Calcified nodule (CN) refers to an atheroma lesion that has protruding nodular calcification penetrating the lumen surface with the attached thrombus.^4^ The CN is regarded as a distinct plaque phenotype with different characteristics.^4^^,^^5^ Despite being well-known that CN prevalence in culprit lesions among ACS patients is the lowest compared to plaque rupture or erosion, existing literature showed a varying prevalence^4^^,^^6^^,^^7^^,^^8^ while its actual prevalence is unknown. The factors associated with CN are also still debatable.

CN may also be associated with worse clinical outcomes than other plaque morphologies. ACS patients with CN exhibit an increased risk of having major adverse cardiovascular events (MACEs), chiefly contributed by the higher rate of ACS recurrence requiring target lesion revascularization.^5^ Indeed, luminal re-protrusion of CN becomes the underlying mechanism of those unfavorable outcomes. However, around 78% of observed in-stent restenosis at CN lesions appears as protruding mass with acoustic shadowing feature, instead of neointimal hyperplasia, suggesting continuous progression after stent implantation.^5^ Similarly, another study emphasizes that the presence of dense calcification with a unique pattern that protrudes to the lumen could affect the clinical outcome following percutaneous coronary intervention (PCI).^9^ The effectivity of modern drug-eluting stents (DESs), one of the known best devices to improve ACS patient outcomes, is also significantly diminished in calcified lesions.^10^^,^^11^ Referring to the exerted primary mechanism of DES, which is by halting hyperplasia of vessel neointima, this could be the reasonable explanation for diminished effectivity of DES in ACS patients with this plaque morphology.^12^ Nevertheless, the exact outcomes of CN are still questionable due to the paucity of clinical data of CN-associated ACS, warranting further investigation and elaboration.^5^^,^^9^^,^^10^^,^^11^

Intravascular imaging, such as optical coherence tomography (OCT), can visualize plaque with a resolution of 10–20 μm.^13^ In the middle of emerging intravascular imaging usage, plaque phenotype can be identified in various parts of the world.^13^ However, the literature that reports CN’s characteristic is limited, with a small sample size and varying results. Hereby, we conducted this systematic review and meta-analysis to pool and analyze the characteristics of CN in ACS from existing literature.

## Results

### Study selection process

**Figure 1:**
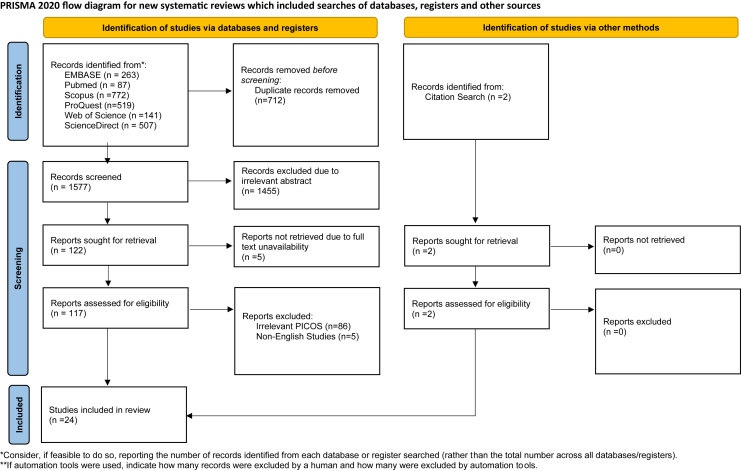
PRISMA 2020 flow diagram for study selection

A comprehensive search was conducted across six databases, resulting in the identification of a total of 2,289 studies (Figure 1). These studies underwent a process of duplicate removal, followed by screening of titles and abstracts, and subsequent full-text assessment. Consequently, 22 studies met the necessary criteria for inclusion and were deemed eligible for further analysis. Additionally, manual searching through citation lists was performed, leading to the discovery of two additional studies meeting the eligibility criteria for inclusion. Therefore, this systematic review and meta-analysis encompassed a total of twenty four studies, involving 15,209 patients diagnosed with ACS associated with CN (Figure 1).

### Quality assessment of included studies

A total of seventeen cohort studies, six cross-sectional studies, and one case-control study were assessed using the NOS quality assessment tool for cohort, cross-sectional, and case-control studies, respectively (refer to Table 1 and Supplementary files: Tables S3, S4, and S5). Overall, 13 studies were considered good, 11 studies were rated fair, and none of the studies were deemed poor.

**Table 1:** Baseline characteristics of included studies

No. Study	Author	Study Location	Study Design		NOS Assessment	Age^a^	Sex (Male)	Prevalence of CN			Culprit Lesion Diagnosis
								Total ACS Patients	CN Patients	Prevalence (%)	
1	Cascon-Perez et al., 2013	Spain	Cross-sectional	–	Fair	N/A	N/A	98	23	23.5	IVUS
2	Jia et al.^12^	Multinational	Cohort	Retrospective	Fair	65.1 ± 5.0	8	104	10	9.6	OCT
3	Higuma et al.^14^	Japan	Cohort	Retrospective	Fair	74.0 (71.0–77.0)	9	111	9	8.1	OCT
4	Wang et al.^15^	USA	Cohort	Prospective	Fair	N/A	N/A	62	2	3.2	OCT
5	Kajander et al.^16^	Multinational	Cohort	Prospective	Fair	N/A	N/A	70	5	7.1	OCT
6	Lee et al.^7^	Japan	Cross-sectional	–	Fair	N/A	15	428	37	8.6	OCT
7	Kobayashi et al.^17^	Japan	Cohort	Prospective	Good	N/A	19	333	21	6.3	OCT
8	Kobayashi et al.^18^	Japan	Cohort	Prospective	Good	71.0 (65.0–77.0)	N/A	479	24	5	OCT
9	Shibuya et al.,^19^ 2019	Japan	Cohort	Prospective	Good	N/A	N/A	483	28	5.8	OCT
10	Sugiyama et al.^20^	Multinational	Cohort	Retrospective	Fair	70.2 ± 11.1	28	1225	36	2.9	OCT
11	Khalifa et al.^21^	Japan	Cross-sectional	–	Fair	77.4 ± 10.7	15	288	34	11.8	OCT
12	Sugane et al., 2020	Japan	Cohort	Retrospective	Good	71.0 ± 10.0	25	657	35	5.3	IVUS
13	Takahata et al.^22^	Japan	Cross-sectional	–	Fair	73.0 (64.0–83.0)	18	297	27	9.1	OCT
14	Gomes et al.^23^	Brazil	Cohort	Retrospective	Good	N/A	N/A	110	13	11.8	OCT
15	Nagasawa et al.^8^	Japan	Cohort	Retrospective	Good	75.3 ± 10.5	41	436	61	14	OCT
16	Nakano et al.^24^	Japan	Case Control	Retrospective	Good	N/A	N/A	74	7	9.5	OCT
17	Nozoe et al.^25^	Japan	Cohort	Retrospective	Good	73.1 ± 10.2	120	5332	167	3.1	IVUS
18	Terada et al.^26^	Japan	Cohort	Retrospective	Good	80.0 (72.0–83.0)	11	156	15	9.6	OCT
19	Torii et al.^10^	USA	Cross-sectional	–	Fair	70.0 ± 12.6	13	524	25	4.8	Histopathology
20	Zhang et al., 2021	Japan	Cross-sectional	–	Fair	N/A	N/A	704	20	2.8	OCT
21	Zhao et al.^27^	China	Cohort	Retrospective	Good	N/A	N/A	408	12	2.9	OCT
22	Lei et al.^28^	China	Cohort	Retrospective	Good	65.9 ± 9.5	37	2593	56	2.2	OCT
23	Li et al.^29^	China	Cohort	Prospective	Good	N/A	N/A	127	8	6.3	OCT
24	Usui et al.^30^	Multinational	Cohort	Retrospective	Good	N/A	N/A	110	3	2.7	OCT

a Mean ± standard deviation or median (minimum-maximum); OCT, Optical coherence tomography; IVUS, Intravascular sonography; N/A Not available.

### Prevalence of CN attributable to culprit lesion among patients presenting with ACS

**Figure 2:**
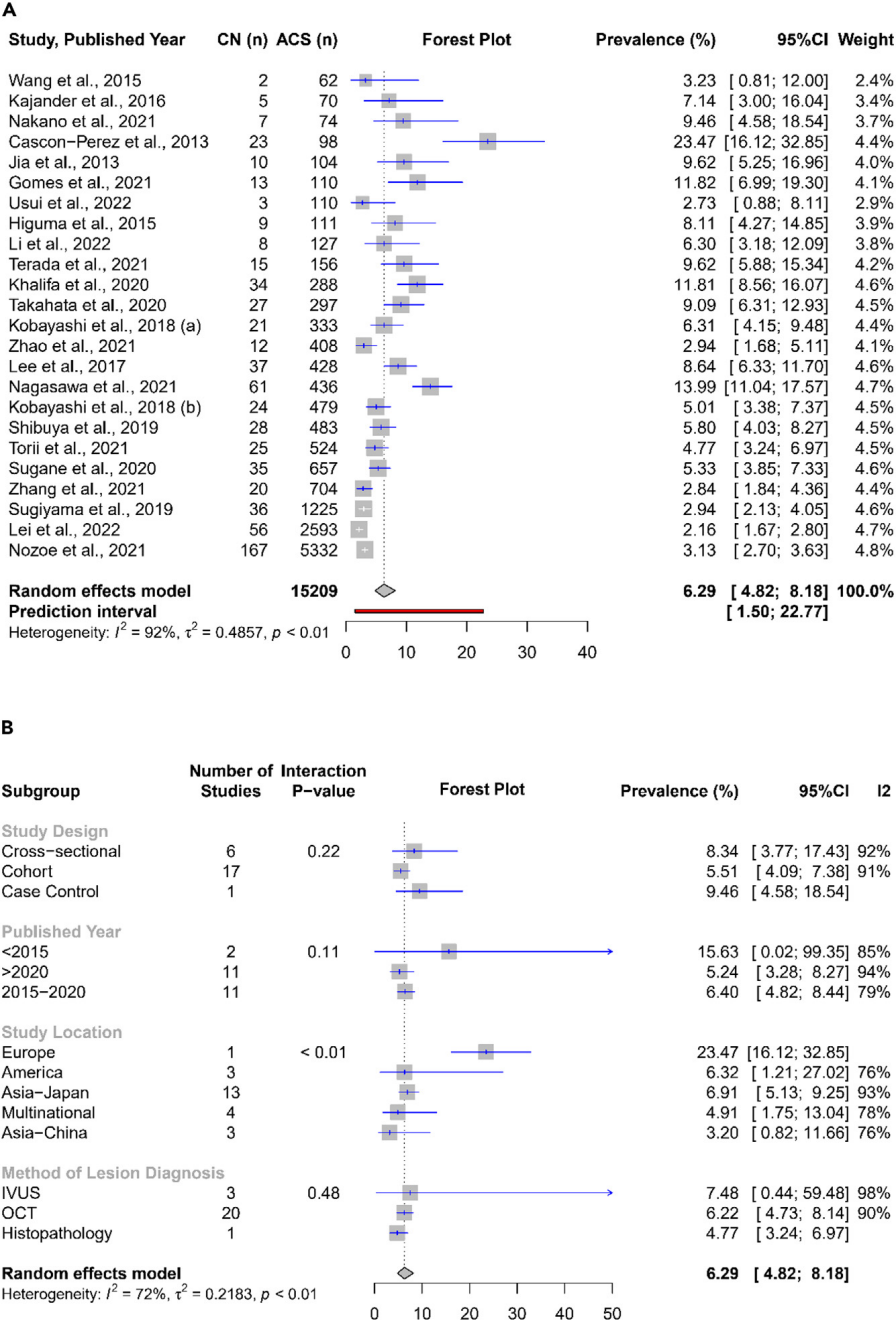
Pooled prevalence of culprit lession-associated CN in ACS patients (A) Pooled prevalence of CN in ACS. (B) Subgroup analysis of pooled prevalence of CN in ACS.

The prevalence of culprit lesion-related CN in patients presenting ACS varied from 2.2% (95% CI 1.7%–2.8%) to 23.5% (95% CI 16.1%–32.9%) in individual studies. The overall pooled prevalence of CN attributable to the culprit lesion was 6.3% (95% CI 4.8%–8.2%), with a significant heterogeneity (I^2^ = 92%) (Figure 2A).

We performed a leave-one-out sensitivity analysis as part of the investigation into heterogeneity (Figure S1). However, excluding any individual study did not lead to a significant reduction in observed heterogeneity. Therefore, we proceeded with identifying possible outliers, defined as studies whose 95% confidence interval falls outside the 95% confidence interval of the pooled effect. Under the assumption of a random effects model, we identified seven outlier studies.^31^^,^^32^^,^^33^^,^^34^^,^^35^^,^^36^^,^^37^ After excluding these outliers, a new pooled estimate was generated, resulting in a prevalence of 6.6% (95% CI 5.4%–8.0%, I2 = 54%, random effects model) (Figure S2).

We subsequently conducted a publication bias analysis on the pooled prevalence of CN in ACS patients. This analysis involved inspecting the generated Funnel’s plot, which displayed the logit-transformed prevalence of each study against its corresponding standard error (Figure S3). Additionally, we employed Egger’s statistical test to quantitatively evaluate the asymmetry of the funnel plot. However, the results of Egger’s test failed to demonstrate the presence of publication bias in this analysis (*p* = 0.09).

Subgroup analysis was planned and carried out as an additional measure to clarify the observed heterogeneity. Study design, published year, study location, NOS quality assessment, and methods of culprit lesion diagnosis, which were determined *a priori*, were subgroups of interest in this review (Figure 2B). However, only the study location subgroups, as we noticed, displayed a significant interaction (*p* < 0.01), indicating the existence of prevalence variation within the study location. A study with participants from the European Continent (Spain) tended to have greater prevalence (23.5% [95% CI 16.1–32.9%]) than studies from all other regions, followed by studies from Japan (6.9% [95% CI 5.1%–9.3%, I^2^ = 93%]).

### Prevalence of CN attributable to culprit lesion across ACS phenotypes

**Figure 3:**
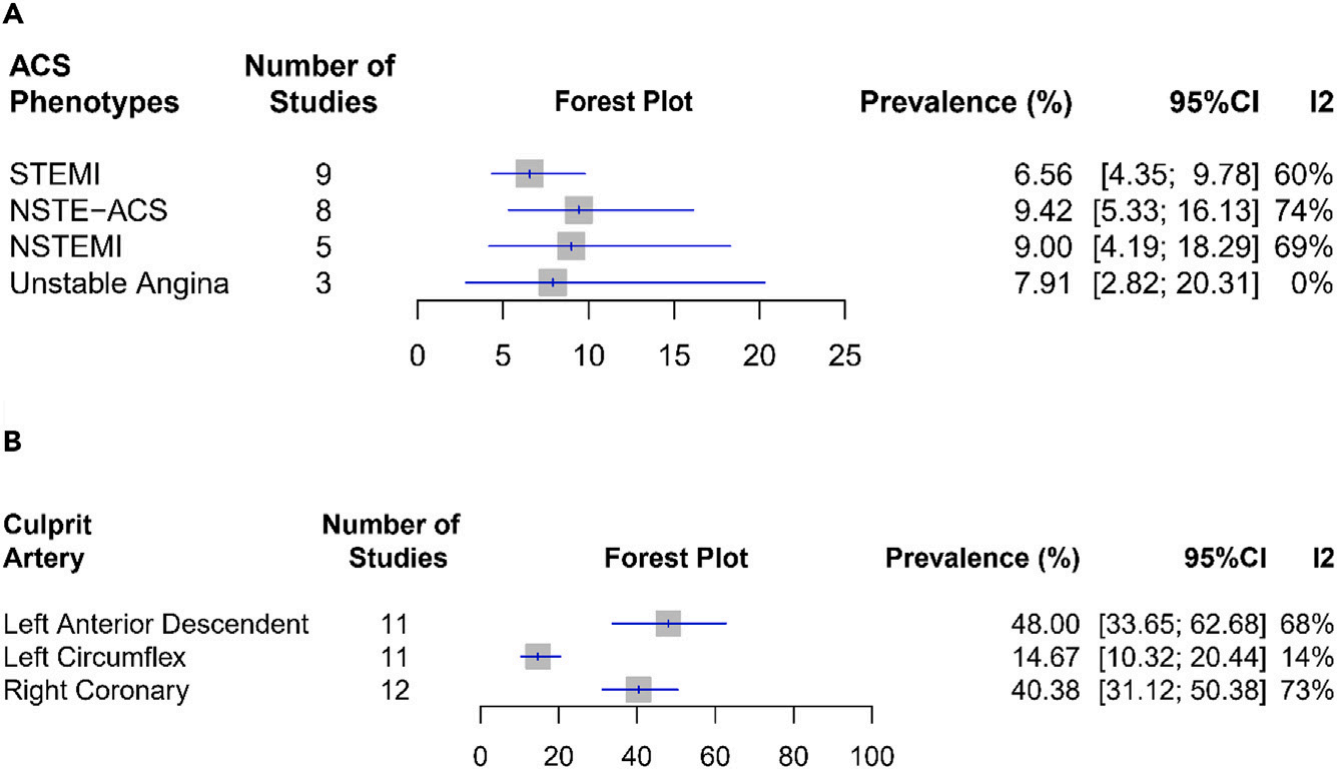
Pooled prevalence of CN in each ACS phenotype and major coronary artery (A) ACS phenotypes. (B) Major coronary arteries.

By aggregating data from nine studies, we found that the prevalence of culprit lesion-related CN in patients with STEMI (*n* = 1631) was 6.6% (95% CI 4.6%–9.8%, I^2^ = 60%), which was the lowest compared to other phenotypes. NSTE-ACS patients (*n* = 966, 8 studies) had the highest prevalence, followed by subphenotype NSTEMI (*n* = 496, 5 studies) and unstable angina (UA) (*n* = 214, 3 studies) patients (Figure 3A). Additionally, regarding the location of culprit arteries associated with CN, 48.0% (95% CI 33.6%–62.7%, I^2^ = 68%) of CN cases were documented in the LAD (*n* = 142/305 patients), followed by RCA (*n* = 12/472), and LCX (*n* = 42/305), respectively (Figure 3B). Detailed information is depicted in Figures 3 and S4–S13.

We conducted a publication bias analysis on the pooled effects of CN’s culprit artery and found possible publication biases in the left circumflex artery (LCX) (Egger’s test *p*-value = 0.03) and right coronary artery (RCA) (Egger’s test *p*-value = 0.01) analyses. Subsequently, we performed trimming and filling analyses and obtained new adjusted estimates for LCX (Figure S14) and RCA on CN (Figure S15), which were 16.7% (95% CI 12.0%–22.8%, I^2^ = 20.1%, random effect) and 50.8% (95% CI 39.1%–62.5%, I^2^ = 78%, random effect), respectively.

### Prevalence of associated cardiovascular comorbidities among patients with calcified and noncalcified nodule

**Figure 4:**
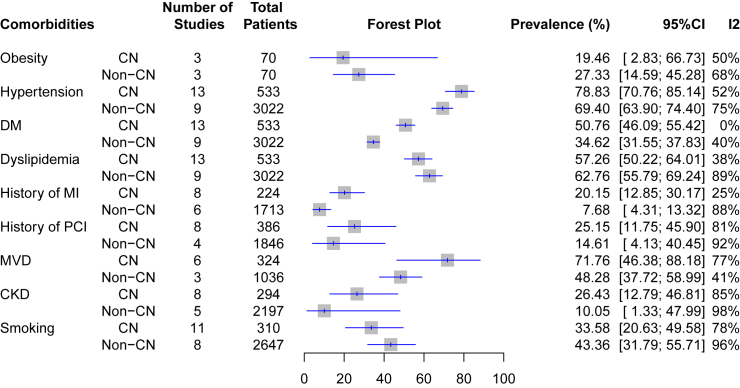
Prevalence of associated cardiovascular comorbidities in CN and non-CN patients

Our analysis of cardiovascular comorbidities revealed a higher prevalence of hypertension, diabetes mellitus, history of myocardial infarction, history of undergoing PCI procedure, evidence of multivessel disease, and chronic kidney disease among patients with CN compared to those without CN (Figure 4, detailed in Figures S5–S13). The substantial prevalence gaps were observed in evidence of multivessel disease (71.8% [46.4%–88.2%] vs. 48.3% [37.7%–60.0%]), followed by diabetes mellitus (50.8% [46.1%–55.4%] vs. 34.6% [31.6%–37.8%]), and chronic kidney disease (26.4% [12.8%–46.8%] vs. 10.1% [1.3%–50.0%]). Since hypertension, diabetes mellitus, dyslipidemia, and smoking prevalence in CN were generated by incorporating the result of more than ten studies, we performed the Egger’s test to assess the presence of publication bias. However, the test results for these variables were statistically insignificant (*p* > 0.05).

### Major cardiac adverse events (MACEs) among patients with CN

**Figure 5:**
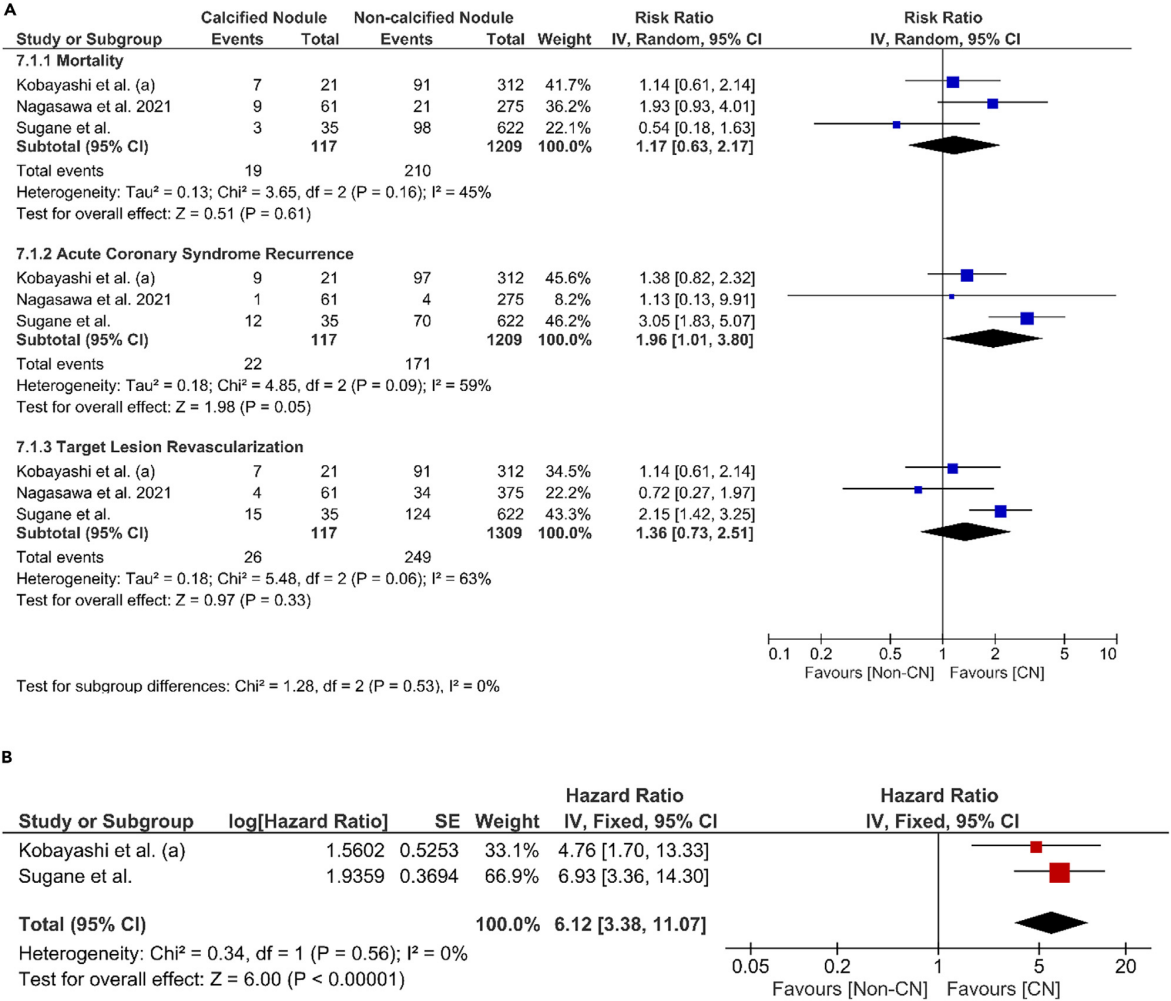
Pooled risk and hazard ratio of mortality, ACS recurrence, and TLR in patients with CN (A) Pooled risk ratio for mortality, ACS recurrence. (B) Pooled hazard ratio of TLR.

Three cohorts have reported the occurrence of MACEs in patients with CN after following them up for specific periods.^5^^,^^8^^,^^17^ We calculated and pooled the risk ratios of mortality, ACS recurrence, and TLR events after a two-year follow-up period (Figure 5A). Our findings indicate that CN did not significantly increase the risk of mortality, ACS recurrence, or target lesion revascularization (TLR) when compared to non-CN lesions. Careful interpretation is warranted due to variations in the reported outcomes among the studies. Specifically, Nagasawa et al. reported all-cause mortality,^8^ whereas the other studies focused on cardiac death. Likewise, Sugane et al. reported ACS recurrence,^5^ while the remaining studies documented myocardial infarction.

Additionally, two cohorts^38^^,^^39^ also reported the adjusted hazard ratio of target lesion revascularization of patients with CN. Our result found that the presence of CN significantly increased the risk of having TLR (HR 6.12, 95% CI 3.38–11.07, I^2^ = 0%) compared to those without CN (Figure 5B). Kobayashi et al.^17^ adjustment included diabetes mellitus, multivessel disease, and DES, with 500 days follow-up periods. Meanwhile, Sugane et al.^5^ adjustment involved diabetes mellitus, estimated glomerular filtration rate, hypertension, major adverse cardiac event (MACE), minimal stent area, and STEMI, with a median of follow-up 1304 days (interquartile range = 846–1747 days).

## Discussion

Despite the global emergence of intracoronary examination techniques, such as OCT, studies reporting on the characteristics of CN remain limited. In this meta-analysis, we meticulously scrutinized 15,209 patients from 24 studies (Table 1) to conduct a pooled analysis, thereby elucidating the defining features of CN. To the best of our knowledge, this first meta-analysis represents the initial endeavor in identifying and delineating the characteristic of CN.

Pooled analysis demonstrated the prevalence of CN is 6.3% (95% CI 4.8%–8.2%) among ACS patients (Figure 2A). That number is quite similar to one of the first large publication CN in literature, around 4.8%.^4^ However, significant heterogeneity was observed (I2 = 92%). Subgroup analysis, then, was the performed even though none of our predetermined subgroups further resulted in significant reductions of heterogeneity. However, we noted that subgroup analysis revealed that non-cohort studies design exhibited a higher prevalence of CN compared to cohort studies (Figure 2B). Cross-sectional studies tend to report a greater number of positive cases than cohort studies. Additionally, we also observed a higher prevalence of CN in studies whose NOS quality assessment results judged fair, published before 2015, employing IVUS technique, and from a European country (Spain). Six out of eleven studies with “fair” NOS judgment were apparently conducted as cross-sectional studies, further supporting previous explanation. We also noticed that published study before 2015 were all judged fair and engaged relatively small sample size. Existence of bias issues and small sample size could lead to the prevalence under- or over-estimation. Small sample size may also introduce the respective study with selection bias related to participation in the study.^40^ Meanwhile, the higher CN prevalence in studies with IVUS as intracoronary imaging still needs to confirmed with future studies though the higher detail provided by OCT offers better identification of luminal lesion, leading to higher specificity in determining the morphological characteristics of CN and subsequent lower false positivity during lesion diagnosis than IVUS.^31^^,^^41^ Besides, IVUS is a highly operator dependent modality which may require extensive experience for applying and interpretation of the results.^42^ Of notes, all of this explanation might contribute to the significantly higher prevalence of CN as the culprit lesion in ACS reported by Cascon-Perez et al. (from Spain), compared to the other studies, as this study was cross-sectional study with small sample size and employing IVUS as the intracoronary imaging modality.

The information regarding the roles of conventional cardiovascular risk factors in CN is scarce. Our analysis demonstrated that not all conventional cardiovascular risk factors had a higher prevalence among CN than non-CN patients (Figure 4). Only hypertension, diabetes mellitus, history of MI, history of PCI, multivessel disease, and CKD had higher prevalence in CN compared to non-CN groups. As a note, we descriptively compared the prevalence of associated comorbidities in CN and non-CN culprit lesions rather than statistically analyzing them. However, the substantial prevalence differences found in multivessel disease (23%), diabetes mellitus (15%), and CKD (15%), which can be considered to contribute to CN formation.

Dense calcification and disruption of the thin fibrous cap are more common in chronic kidney disease.^7^^,^^13^ The previous study conducted by Okamura et al.^43^ reported CN culprit lesion prevalence in the CKD population with ACS is ∼25%.^44^ Renal dysfunction causes abnormal calcium and phosphor metabolism, and inflammatory cytokine and calcification-related protein secretion that seem to contribute to atheromatous plaque calcification and might serve as crucial factors contributing to the development of CNs.^45^ A study by Nakamura added that in-stent CNs are most common cause of restenosis and observed within 1 year after stent implantation in CKD patients undergoing hemodialysis.^46^ Recent review also collected information that combination of mineral metabolism and increased phosphate, fibroblast growth factor 23, parathyroid hormone, and 1,25-dihydroxy vitamin D, and local and systemic factors could contribute to cardiovascular calcification in CKD.^32^^,^^47^ In pre-dialysis and late-stage CKD patients, calcification of the coronary artery reached 64–77%.^48^^,^^49^^,^^50^ However, the mechanisms through which CKD result in cardiovascular calcification appears complex and is still being investigated.^47^ Eventually, we do not include Okamura et al.^43^ study in this our pooled analysis (Figure 1) as it reflects CN prevalence in a specific population.

Another study also demonstrated CN is more prevalent in diabetes mellitus.^7^ Alongside CKD, diabetes mellitus promotes both intimal and medial calcification, with a notably significant influence observed in the latter layer.^51^^,^^52^ Intimal calcification has been associated with an elevated risk of CN rupture, while medial calcification promotes arterial stiffness, resulting in hypertension, as evidenced in our findings which show a higher prevalence of hypertension in CN group.^33^^,^^53^ These findings lend support to the hypothesis that there is a higher prevalence of CKD and diabetes mellitus in populations with CN, suggesting them as potential risk factors for CN formation. Similar observations may be made in cases of multivessel disease. In diabetic patients, coronary artery disease tends to manifest as a more complex condition characterized by small, diffuse, and calcified lesions, often involving multiple vessels.^34^^,^^54^ Furthermore, a study by Sugane et al. summarized that patients with CN have an increased risk of ACS recurrence, including those with MI, due to the continuous growth and protrusion of the calcified mass.^5^ ACS recurred more often in individuals with CNs, primarily due to in-stent restenosis (ISR). In lesions with CN, the rates of in-stent restenosis (ISR) were also significantly higher compared to those without CN (43.8% vs. 25.0%, *p* = 0.023).^38^ This might explain the reason for the high history of previous PCI or MI among patients with CN. Besides, the high prevalence of diabetes mellitus, hypertension, and CKD may predispose patients with these risk factors to have MVD and recurrent MI, with or without PCI.^35^^,^^36^^,^^55^^,^^56^

In addition, above explanation also provides an alternative explanation for the substantial disparities in CN prevalence reported by Cascon-Perez from Spain, discussed before, as the potential risk factors associated with CN and the prevalence of metabolic syndrome were commonly observed in the European population.^57^ Both the Western dietary pattern and ethnic predisposition may contribute to the higher prevalence of CN observed in that study. A study observed that coronary calcification was highest in the white population (70.4%), followed by the Chinese (59.2%), Hispanic (56.5%), and Black (52.1%) populations.^39^ The Western dietary pattern, characterized by increased intake of fat, red meat, and carbohydrates, and minimal consumption of fruits and green leafy vegetables, is also known to be associated with severe coronary artery disease.^58^ Nevertheless, further research is warranted to confirm these findings.

While referring to the current ACS classification,^37^^,^^59^ the prevalence of CN in STEMI is lower than in NSTE-ACS (6.6% vs. 9.4%) (Figure 3A). This observation is in concordance with a previous study that demonstrated a higher CN proportion in NSTE-ACS than STEMI.^12^ Another study showed that CN prevalence in NSTE-ACS is much lower than STEMI (2.9% vs. 8%).^60^ However, the mentioned study has a retrospective cross-sectional design and unbalanced participants between STEMI and NSTE-ACS, thus increasing the risk of statistical errors.^23^ The possible explanation, however, may align with the previous discussions regarding the higher prevalence of diabetes, MVD, and hypertension being associated with CN. NSTE-ACS occurs as a result of myocardial ischemia and injury, often resulted from partial or intermittent occlusion along the ischemic cascade.^60^ Notably, NSTE-ACS also presents more commonly in patients with diabetes and renal dysfunction within the ACS population.^44^^,^^61^ Meanwhile, MVD is a well-established common finding in NSTE-ACS patients.^62^^,^^63^ We propose that a complex interplay involving diabetes, hypertension, and renal dysfunction underlies the higher prevalence of MVD and, subsequently, the NSTE-ACS presentation in patients with CN. However, irrespective of the classification of ACS, the prevalence of CN in culprit lesions remains below 10% (Figure 4).

Interestingly, the pooled analysis revealed a higher prevalence of CN in LAD and RCA, while the lowest prevalence was observed in the LCX (48%, 40%, and 15%, respectively) (Figure 3B). Maximal torsion in the mid-part of the right coronary artery and left anterior descending artery^4^^,^^7^ may contribute to differences in CN prevalence according to the epicardial vessel. These nodules, in general, are typically more frequently found in proximal or mid vessels and may also be associated with diabetes which tends to affect these areas. Additionally, hypertension may exert pulsatile mechanical stress on plaques,^51^ potentially contributing to a similar pathophysiological mechanism underlying the occurrence of CN eruption.

This meta-analysis also demonstrated that CN lesion is associated with TLR (aHR 6.1, 95% CI 3.4–11.1) (Figure 5). CN lesion is characterized by dense calcification that causes stent under-expansion and incomplete apposition^21^ which are known as predictors of TLR. CN re-protrusion occurs even after stent implantation and is observed in 78% of in-stent restenosis^5^ which may contribute to higher TLR in CN plaque morphology. In addition, the effectivity of DES also diminished as outcomes improvement in DES usage was primarily attained by restricting neointimal hyperplasia which is not the main atherothrombotic mechanism in CN.^12^ This was also supported by our pooled finding that prevalence or previous MI and PCI were higher in patients with compared to without CN (Figure 4), which may be due to the higher occurrence of ACS recurrence and TLR (Figure 5). However, this finding warrants careful interpretation as the pooled estimate was drawn from only two cohorts and future robust studies involving larger sample sizes are still needed to confirm this association.

### Limitations of the study

There are several limitations to this meta-analysis. First, it is important to acknowledge that the included studies in this meta-analysis employ different observational designs, which may introduce the possibility of bias due to variations in methodologies and selection processes. However, to anticipate this potential bias, subgroup analysis was conducted revealing no significant differences in prevalence among the various study designs. All studies employed consecutive sampling technique, which may also contribute to the persistent heterogeneities in some analyses. Additionally, it is worth noting that the majority (75%) of the study designs in this meta-analysis are cohort studies, which offer more robust data and risk estimation compared to other observational study designs. Second, as the pooled studies were majorly from the Asian population, it might limit the generalizability of our findings. Third, it is crucial to recognize that the prevalence of each associated cardiovascular risk factor in CN and non-CN lesions was described descriptively rather than being directly compared through statistical analysis. While the descriptive statistics indicate a higher proportion of CN lesions compared to non-CN lesions, it is a must to perform statistical analysis, particularly multivariate analysis, to accurately determine the contribution of each cardiovascular risk factor to CN formation. Fourth, the outcome analysis in this meta-analysis is based on a limited number of studies, with only three studies included, each having small sample sizes and varying follow-up periods. This insufficiency in the number of studies and the variations in sample sizes and follow-up periods may restrict the precise estimation of outcomes in CN.

Despite these aforementioned limitations, significant efforts were made to anticipate bias in this meta-analysis. It is crucial to emphasize that this study represents the first systematic review and meta-analysis that consolidates data from published articles to define the characteristics of CN in culprit lesions among ACS patients. By providing these characteristics, this meta-analysis serves as a valuable resource for both clinical and basic research purposes. Our findings also shed a light on the needs for a different approach in managing ACS patients with CN as the culprit lesion since the common management strategies might not be effective for this entity. However, it is important to conduct well-designed studies with larger sample sizes to validate the findings of this meta-analysis.

### Conclusion

The CN is a distinct phenotype of an atheromatous plaque, accounting for 6.3% of ACS cases. Regardless of the type of ACS and the diagnostic modality employed, the prevalence of CN remains below 10%. However, it is important to note that the prevalence of hypertension, multivessel disease, diabetes mellitus, and chronic kidney disease may be higher in individuals with CN attributable to ACS. Notably, ACS cases associated with CN may exhibit an increased likelihood of requiring target lesion revascularization. Well-designed studies are still required to confirm these findings and provide additional information to complement the results of this meta-analysis.

## STAR★Methods

### Key resources table

**Table:** 

REAGENT or RESOURCE	SOURCE	IDENTIFIER
**Deposited data**		

International Prospective Register of Systematic Reviews	https://www.crd.york.ac.uk/prospero/	PROSPERO
EMBASE	https://www.embase.com/	N/A
PubMed	https://pubmed.ncbi.nlm.nih.gov/advanced/	N/A
ProQuest	https://www.proquest.com/	N/A
Scopus	https://www.scopus.com/search/form.uri?display=advanced	N/A
Web of Science	https://www.webofscience.com/wos/woscc/advanced-search	N/A
ScienceDirect	https://www.sciencedirect.com/search/entry	N/A

**Software and algorithms**		

R software in RStudio	https://posit.co/download/rstudio-desktop/	Version 4.2.2
Review Manager	https://training.cochrane.org/online-learning/core-software/revman	Version 5.4.1

### Resource availability

#### Lead contact

For additional information and resources, they should be directed to the lead contact, Dr. Citrawati Dyah Kencono Wungu, MD., Ph.D. (citrawati.dyah@fk.unair.ac.id).

#### Materials availability

This study is a systematic review and meta-analysis. Therefore, it did not use or generate any reagents.

#### Data and code availability

• All data are available in the article and/or supplementary files.
• Any additional information required to reanalyze the data reported in this paper is available from the lead contact upon request.
• This study did not report the original code.
• All information regarding searched databases and used software are listed in key resources table.

### Experimental model and study participant details

#### Experimental model

As this study is a systematic review and meta-analysis, it does not use experimental models typical in the life sciences.

#### Subject details

This review engaged subjects from a total of 24 observational studies, comprising of 17 cohort, 6 cross-sectional. Four studies were multinational,^13^^,^^16^^,^^20^^,^^30^ while three studies were from China,^27^^,^^28^^,^^29^ two studies were from the United States of America,^10^^,^^15^ one study was from Brazil,^23^ one study was from Spain,^6^ and the remaining thirteen were originated from Japan^5^^,^^7^^,^^8^^,^^17^^,^^21^^,^^14^^,^^18^^,^^19^^,^^22^^,^^24^^,^^25^^,^^26^^,^^64^ (Table 1). All included studies employed consecutive sampling, even though samples from Wang et al. was from a randomized clinical trial assessing the comparison of manual thrombus aspiration and rheolytic thrombectomy in acute myocardial infarction.^15^ Male patients constituted the majority in most of the included studies, while the mean and median ages of the patients consistently exceeded 65 years old. Optical coherence tomography (OCT) was selected as the primary diagnostic tool for lesion diagnosis in most studies. However, it is worth noting that three studies employed intravascular ultrasonography (IVUS),^5^^,^^6^^,^^25^ and one study relied on histopathology examinations.^10^ We did not performed specific analysis in regard to ancestry, race or ethnicity of included patients.

### Method details

We followed the guidelines provided by the Preferred Reporting Items for Systematic Review and Meta-analysis (PRISMA) to conduct this systematic review (see Supporting Information: Table S1). To ensure transparency and adherence to standard practices, we created a review protocol for this study and registered it in the International Prospective Register of Systematic Reviews database (PROSPERO CRD42023406609).

#### Search Strategy

We systematically searched published literature in multiple databases (EMBASE, PubMed, ProQuest, Scopus, Web of Science, and ScienceDirect) from their inception dates until March 14, 2023. In addition, we conducted hand-searching through citation snowballing to broaden the scope of our search. The search terms and strategy were determined *a priori*, which were (“acute coronary syndrome” OR “STEMI” OR “NSTEMI” OR “NSTE-ACS” OR “unstable angina”) AND (“nodular calcification” OR “calcified nodule”) (Refers to Table S2). The titles and abstracts yielded from database searches were independently screened to determine their eligibility. Subsequently, studies with eligible abstracts were reviewed for full-text articles based on predetermined inclusion and exclusion criteria. Any disagreements among the authors were resolved through consensus, which involved senior authors.

#### Eligibility criteria

The present review included observational studies of the following types: cross-sectional, case-control, and cohort studies. The screening of eligible studies was in accordance with specific inclusion criteria, which were as follows: (1) inclusion of patients of all ages with an acute coronary syndrome, per the definition provided by the European Society of Cardiology (ESC) guidelines^37^^,^^59^; (2) undergoing optical coherence tomography (OCT), intravascular ultrasonography (IVUS), or histopathology examination; and (3) reporting at least the prevalence of CN, pathologically defined as lesions with fibrous cap disruption and luminal thrombus associated with eruptive, dense, calcific nodules.^4^ In. addition, CN according to OCT defined as a fibrous cap disruption over a calcified plaque and is characterized by protruding calcification, superficial and substantive calcium.^65^ Further, It is identified with IVUS as convex or irregular formations with severe acoustic "shadowing" often protruding into the vessel lumen.^66^ Studies that involved fewer than fifty ACS patients, reported CN on non-culprit lesions, reported calcified plaques without a specific number of CN, or were delivered in a non-English language were excluded.

#### Data extraction

Three reviewers independently performed the selection of studies and subsequent extraction of data. The extracted data were tabulated in a Google Spreadsheet (Google LLC, USA). The following information was documented: study characteristics (first author, publication year, study location, study design, study inclusion and exclusion criteria), baseline information of patients (age, sex), prevalence of CN (number of patients with CN-associated culprit lesions, total ACS patients), location of culprit lesions (left anterior descending artery [LAD], left circumflex artery [LCX], right coronary artery [RCA]), associated cardiovascular comorbidities (obesity, hypertension, diabetes mellitus, dyslipidemia, history of myocardial infarction [MI], history of undergoing percutaneous coronary intervention [PCI], presence of multivessel disease, chronic kidney disease or eGFR<60, and smoking) among patients with CN or non-CN, and the Major Adverse Cardiovascular Events (MACE) outcomes of patients after experiencing CN-associated ACS (including risk and hazard ratio, follow-up period for composite MACE, cardiac death, myocardial infarction, ACS recurrence, target lesion revascularization, and mortality).

### Quantification and statistical analysis

#### Quality assessment

We used the Newcastle-Ottawa Scale (NOS) Quality Assessment Tool for assessing the quality of included studies (cohort, cross-sectional, and case-control studies).^67^ The NOS offers a standardized methodology for evaluating the quality of non-randomized studies based on three domains: research group selection, group comparability, and identification of the relevant exposure/outcome. NOS gives each study a score of up to a maximum of nine points. Studies with ratings of at least seven are deemed to be of "Good" quality, studies with scores of 5–6 are deemed to be "Fair", and studies with scores of less than 5 are deemed to be of "Poor" quality.^68^ Any discrepancies were resolved by discussion between the authors.

#### Statistical analysis

Data were summarized and presented descriptively in tabular format and quantitatively through meta-analysis of prevalence and ratio. The meta-analysis was done with the "meta", "metafor" and "dmetar" packages in R software version 4.2.2 (Posit PBC, USA) and Review Manager version 5.4 (Cochrane Collaboration, UK). A forest plot was used to visualize all pooled prevalence and ratios. Random effects meta-analysis was used to anticipate between-study heterogeneity. Pooled prevalence and ratios were estimated using the inverse of the variance of the logit-transformed proportion and ratio measures (risk or hazard ratio), respectively. We used the I^2^ test to quantify the heterogeneity between studies, with values of I^2^ > 50% representing moderate-to-high heterogeneity.^43^ For the general prevalence of CN attributable to the culprit lesion among ACS patients, we conducted subgroup analyses according to published year, study location, study design, and methods of lesion diagnosis to try to elucidate the potential source of between-study heterogeneities. Sensitivity analyses were also performed through leave-one-out sensitivity analysis and outliers study exclusion (studies whose 95% confidence interval falls outside the 95% confidence interval of the pooled effect) to demonstrate how each study influenced the overall result. Publication bias analyses were tested both visually by Funnel’s plot and statistically by Egger’s regression test.^69^ Whenever significant publication bias existed, we conducted the Duval and Tweedie trim and fill method to generate adjusted effect estimates.^70^ All statistical analyses with a *p*-value less than 0.05 were considered significant.

#### Additional resources

All data in this study were collected from the included literature and do not require ethical approval.

## Supplemental information

Document S1. Figures S1–S15

Table S1. PRISMA 2020 checklist, related to method details

Table S2. Detailed search strategy, related to search strategy in STAR Methods

Table S3. NOS quality assessment tool for observational cohort studies, related to quality assessment in STAR Methods

Table S4. NOS quality assessment tool for observational cross-sectional studies, related to quality assessment in STAR Methods

Table S5. NOS quality assessment tool for observational case-control studies, related to quality assessment in STAR Methods

## References

[1] Sanchis-Gomar F., Perez-Quilis C., Leischik R., Lucia A. (2016). Epidemiology of coronary heart disease and acute coronary syndrome. Ann. Transl. Med..

[2] Mozaffarian D., Benjamin E.J., Go A.S., Arnett D.K., Blaha M.J., Cushman M., De Ferranti S., Després J.P., Fullerton H.J., Howard V.J. (2015). Heart Disease and Stroke Statistics—2015 Update: A Report from the American Heart Association. Circulation.

[3] Bentzon J.F., Otsuka F., Virmani R., Falk E. (2014). Mechanisms of plaque formation and rupture. Circ. Res..

[4] Virmani R., Kolodgie F.D., Burke A.P., Farb A., Schwartz S.M. (2000). Lessons From Sudden Coronary Death A Comprehensive Morphological Classification Scheme for Atherosclerotic Lesions. Arterioscler. Thromb. Vasc. Biol..

[5] Sugane H., Kataoka Y., Otsuka F., Nakaoku Y., Nishimura K., Nakano H., Murai K., Honda S., Hosoda H., Matama H. (2021). Cardiac outcomes in patients with acute coronary syndrome attributable to calcified nodule. Atherosclerosis.

[6] Cascón-Pérez J.D., De La Torre-Hernández J.M., Ruiz-Abellón M.C., Martínez-Pascual M., Mármol-Lozano R., López-Candel J., Cano P., Fernández C., Ramos J.L., Villegas M., Picó-Aracil F. (2013). Characteristics of culprit atheromatous plaques obtained in vivo by intravascular ultrasound radiofrequency analysis: Results from the CULPLAC study. Am. Heart J..

[7] Lee T., Mintz G.S., Matsumura M., Zhang W., Cao Y., Usui E., Kanaji Y., Murai T., Yonetsu T., Kakuta T., Maehara A. (2017). Prevalence, Predictors, and Clinical Presentation of a Calcified Nodule as Assessed by Optical Coherence Tomography. JACC. Cardiovasc. Imaging.

[8] Nagasawa A., Otake H., Kawamori H., Toba T., Sugizaki Y., Takeshige R., Nakano S., Tanimura K., Takahashi Y., Fukuyama Y. (2021). Relationship among clinical characteristics, morphological culprit plaque features, and long-term prognosis in patients with acute coronary syndrome. Int. J. Cardiovasc. Imaging.

[9] Kobayashi Y., Okura H., Kume T., Yamada R., Kobayashi Y., Fukuhara K., Koyama T., Nezuo S., Neishi Y., Hayashida A. (2014). Impact of target lesion coronary calcification on stent expansion. Circ. J..

[10] Torii S., Sato Y., Otsuka F., Kolodgie F.D., Jinnouchi H., Sakamoto A., Park J., Yahagi K., Sakakura K., Cornelissen A. (2021). Eruptive Calcified Nodules as a Potential Mechanism of Acute Coronary Thrombosis and Sudden Death. J. Am. Coll. Cardiol..

[11] Nishida K., Nakatsuma K., Shiomi H., Natsuaki M., Kawai K., Morimoto T., Kozuma K., Igarashi K., Kadota K., Tanabe K. (2018). Second-generation vs. First-generation drug-eluting stents in patients with calcified coronary lesions: Pooled analysis from the reset and next trials. Circ. J..

[12] Otsuka F., Byrne R.A., Yahagi K., Mori H., Ladich E., Fowler D.R., Kutys R., Xhepa E., Kastrati A., Virmani R., Joner M. (2015). Neoatherosclerosis: Overview of histopathologic findings and implications for intravascular imaging assessment. Eur. Heart J..

[13] Jia H., Abtahian F., Aguirre A.D., Lee S., Chia S., Lowe H., Kato K., Yonetsu T., Vergallo R., Hu S. (2013). In vivo diagnosis of plaque erosion and calcified nodule in patients with acute coronary syndrome by intravascular optical coherence tomography. J. Am. Coll. Cardiol..

[14] Higuma T., Soeda T., Abe N., Yamada M., Yokoyama H., Shibutani S., Vergallo R., Minami Y., Ong D.S., Lee H. (2015). A Combined Optical Coherence Tomography and Intravascular Ultrasound Study on Plaque Rupture, Plaque Erosion, and Calcified Nodule in Patients With ST-Segment Elevation Myocardial Infarction Incidence, Morphologic Characteristics, and Outcomes After Percutaneous Coronary Intervention. http://www.acc.or/jacc-journals-cme.

[15] Wang L., Parodi G., Maehara A., Valenti R., Migliorini A., Vergara R., Carrabba N., Mintz G.S., Antoniucci D. (2015). Variable underlying morphology of culprit plaques associated with ST-elevation myocardial infarction: An optical coherence tomography analysis from the SMART trial. Eur. Heart J. Cardiovasc. Imaging.

[16] Kajander O.A., Pinilla-Echeverri N., Jolly S.S., Bhindi R., Huhtala H., Niemelä K., Fung A., Vijayaraghavan R., Alexopoulos D., Sheth T. (2016). Culprit plaque morphology in STEMI-an optical coherence tomography study: Insights from the TOTAL-OCT substudy. EuroIntervention..

[17] Kobayashi N., Takano M., Tsurumi M., Shibata Y., Nishigoori S., Uchiyama S., Okazaki H., Shirakabe A., Seino Y., Hata N., Shimizu W. (2018). Features and Outcomes of Patients with Calcified Nodules at Culprit Lesions of Acute Coronary Syndrome: An Optical Coherence Tomography Study. Cardiology.

[18] Kobayashi N., Hata N., Tsurumi M., Shibata Y., Okazaki H., Shirakabe A., Takano M., Seino Y., Shimizu W. (2018). Relation of Coronary Culprit Lesion Morphology Determined by Optical Coherence Tomography and Cardiac Outcomes to Serum Uric Acid Levels in Patients With Acute Coronary Syndrome. Am. J. Cardiol..

[19] Shibuya J., Kobayashi N., Asai K., Tsurumi M., Shibata Y., Uchiyama S., Okazaki H., Goda H., Tani K., Shirakabe A. (2019). Comparison of Coronary Culprit Lesion Morphology Determined by Optical Coherence Tomography and Relation to Outcomes in Patients Diagnosed with Acute Coronary Syndrome During Winter –vs– Other Seasons. Am. J. Cardiol..

[20] Sugiyama T., Yamamoto E., Fracassi F., Lee H., Yonetsu T., Kakuta T., Soeda T., Saito Y., Yan B.P., Kurihara O. (2019). Calcified Plaques in Patients With Acute Coronary Syndromes. JACC Cardiovasc. Interv..

[21] Khalifa A.K.M., Kubo T., Ino Y., Terada K., Emori H., Higashioka D., Katayama Y., Takahata M., Shimamura K., Shiono Y. (2020). Optical coherence tomography comparison of percutaneous coronary intervention among plaque rupture, erosion, and calcified nodule in acute myocardial infarction. Circ. J..

[22] Takahata M., Ino Y., Kubo T., Tanimoto T., Taruya A., Terada K., Emori H., Higashioka D., Katayama Y., Khalifa A.K.M. (2020). Prevalence, features, and prognosis of artery-to-artery embolic st-segment– elevation myocardial infarction: An optical coherence tomography study. J. Am. Heart Assoc..

[23] Gomes P.M., Almeida B.O., Marinelli Pedrini S., Freitas B.P., Júnior J.M., Lemos P.A., Fonseca F.H., Mintz G.S., Caixeta A. (2021). Morphology and phenotype characteristics of atherosclerotic plaque in patients with acute coronary syndrome: Contemporary optical coherence tomography findings. Coron. Artery Dis..

[24] Nakano S., Otake H., Kawamori H., Toba T., Sugizaki Y., Nagasawa A., Takeshige R., Matsuoka Y., Tanimura K., Takahashi Y. (2021). Association Between Visit-to-Visit Variability in Low-Density Lipoprotein Cholesterol and Plaque Rupture That Leads to Acute Coronary Syndrome. Circ. Rep..

[25] Nozoe M., Nishioka S., Oi K., Suematsu N., Kubota T. (2021). Effects of Patient Background and Treatment Strategy on Clinical Outcomes After Coronary Intervention for Calcified Nodule Lesions. Circ. Rep..

[26] Terada K., Kubo T., Kameyama T., Matsuo Y., Ino Y., Emori H., Higashioka D., Katayama Y., Khalifa A.K.M., Takahata M. (2021). NIRS-IVUS for Differentiating Coronary Plaque Rupture, Erosion, and Calcified Nodule in Acute Myocardial Infarction. JACC. Cardiovasc. Imaging.

[27] Zhao L., Du Z., Wu T., Cao M., Wang Y., Zhao J., Dong H., Wang C., Jia H., Yu B. (2021). Association of the age shock index with coronary plaque characteristics in ST-segment elevation myocardial infarction: A 3-vessel optical coherence tomography study. Catheter. Cardiovasc. Interv..

[28] Lei F., Yin Y., Liu X., Fang C., Jiang S., Xu X., Sun S., Pei X., Jia R., Tang C. (2022). Clinical Outcomes of Different Calcified Culprit Plaques in Patients with Acute Coronary Syndrome. J. Clin. Med..

[29] Li J., Chen R., Zhou J., Wang Y., Zhao X., Liu C., Zhou P., Chen Y., Song L., Yan S. (2022). Prognostic Value of Admission Peak NT-proBNP Combined with Culprit Plaque Types for Predicting Cardiovascular Risk in ST-Segment Elevated Myocardial Infarction: An Optical Coherence Tomography Study. J. Cardiovasc. Dev. Dis..

[30] Usui E., Matsumura M., Smilowitz N.R., Mintz G.S., Saw J., Kwong R.Y., Hada M., Mahmud E., Giesler C., Shah B. (2022). Coronary morphological features in women with non-ST-segment elevation MINOCA and MI-CAD as assessed by optical coherence tomography. Eur. Heart J. Open.

[31] Nagaraja V., Kalra A., Puri R. (2020). When to use intravascular ultrasound or optical coherence tomography during percutaneous coronary intervention?. Cardiovasc. Diagn. Ther..

[32] (2017). Erratum: Kidney Disease: Improving Global Outcomes (KDIGO) CKD-MBD Update Work Group. KDIGO 2017 Clinical Practice Guideline Update for the Diagnosis, Evaluation, Prevention, and Treatment of Chronic Kidney Disease–Mineral and Bone Disorder (CKD-MBD). Kidney Int. Suppl..

[33] London G.M., Marchais S.J., Guerin A.P. (2004). Arterial stiffness and function in end-stage renal disease. Adv. Chronic Kidney Dis..

[34] Creager M.A., Lüscher T.F., Cosentino F., Beckman J.A. (2003). Diabetes and vascular disease. Pathophysiology, clinical consequences, and medical therapy: Part I. Circulation.

[35] Picariello C., Lazzeri C., Attanà P., Chiostri M., Gensini G.F., Valente S. (2011). The impact of hypertension on patients with acute coronary syndromes. Int. J. Hypertens..

[36] Serhiyenko V.A., Serhiyenko A.A. (2021). Diabetes mellitus and acute coronary syndromes. Int. J. Endocrinol..

[37] Ibanez B., James S., Agewall S., Antunes M.J., Bucciarelli-Ducci C., Bueno H., Caforio A.L.P., Crea F., Goudevenos J.A., Halvorsen S. (2018). 2017 ESC Guidelines for the management of acute myocardial infarction in patients presenting with ST-segment elevation: The Task Force for the management of acute myocardial infarction in patients presenting with ST-segment elevation of the European Society of Cardiology (ESC). Eur. Heart J..

[38] Tada T., Miura K., Ikuta A., Ohya M., Shimada T., Osakada K., Takamatsu M., Taguchi Y., Kubo S., Tanaka H. (2022). Prevalence, predictors, and outcomes of in-stent restenosis with calcified nodules. EuroIntervention..

[39] Bild D.E., Detrano R., Peterson D., Guerci A., Liu K., Shahar E., Ouyang P., Jackson S., Saad M.F. (2005). Ethnic differences in coronary calcification: The Multi-Ethnic Study of Atherosclerosis (MESA). Circulation.

[40] Buitrago-Garcia D., Salanti G., Low N. (2022). Studies of prevalence: how a basic epidemiology concept has gained recognition in the COVID-19 pandemic. BMJ Open.

[41] Naghavi M., Wang H., Lozano R., Davis A., Liang X., Zhou M., Vollset S.E., Abbasoglu Ozgoren A., Abdalla S., Abd-Allah F. (2015). Global, regional, and national age-sex specific all-cause and cause-specific mortality for 240 causes of death, 1990-2013: A systematic analysis for the Global Burden of Disease Study 2013. Lancet.

[42] Hazan E., Ugurlu B., Sariosmanoglu O., Kozan O., Metin K., Oto O. (2004).

[43] Okamura A., Okura H., Iwai S., Sakagami A., Kamon D., Hashimoto Y., Ueda T., Soeda T., Watanabe M., Saito Y. (2022). Incidence and prognostic impact of the calcified nodule in coronary artery disease patients with end-stage renal disease on dialysis. Heart Ves..

[44] Franczyk-Skóra B., Gluba A., Banach M., Rysz J. (2013). Treatment of non-ST-elevation myocardial infarction and ST-elevation myocardial infarction in patients with chronic kidney disease. Arch. Med. Sci..

[45] Iwai T., Kataoka Y., Otsuka F., Asaumi Y., Nicholls S.J., Noguchi T., Yasuda S. (2019). Chronic kidney disease and coronary atherosclerosis: evidences from intravascular imaging. Expert Rev. Cardiovasc Ther..

[46] Nakamura N., Torii S., Tsuchiya H., Nakano A., Oikawa Y., Yajima J., Nakamura S., Nakano M., Masuda N., Ohta H. (2020). Formation of calcified nodule as a cause of early in-stent restenosis in patients undergoing dialysis. J. Am. Heart Assoc..

[47] Hutcheson J.D., Goettsch C. (2023). Cardiovascular Calcification Heterogeneity in Chronic Kidney Disease. Circ. Res..

[48] Tomiyama C., Carvalho A.B., Higa A., Jorgetti V., Draibe S.A., Canziani M.E.F. (2010). Coronary calcification is associated with lower bone formation rate in CKD patients not yet in dialysis treatment. J. Bone Miner. Res..

[49] Kestenbaum B.R., Adeney K.L., Boer I.H.D., Ix J.H., Shlipak M.G., Siscovick D.S. (2009). Incidence and progression of coronary calcification in chronic kidney disease: The multi-ethnic study of atherosclerosis. Kidney Int..

[50] Piers L.H., Touw H.R.W., Gansevoort R., Franssen C.F.M., Oudkerk M., Zijlstra F., Tio R.A. (2009). Relation of Aortic Valve and Coronary Artery Calcium in Patients With Chronic Kidney Disease to the Stage and Etiology of the Renal Disease. Am. J. Cardiol..

[51] Kalra S.S., Shanahan C.M. (2012). Vascular calcification and hypertension: Cause and effect. Ann. Med..

[52] Van Popele N.M., Grobbee D.E., Bots M.L., Asmar R., Topouchian J., Reneman R.S., Hoeks A.P., van der Kuip D.A., Hofman A., Witteman J.C. (2001). Association Between Arterial Stiffness and Atherosclerosis The Rotterdam Study. Stroke.

[53] Abedin M., Tintut Y., Demer L.L. (2004). Vascular calcification: Mechanisms and clinical ramifications. Arterioscler. Thromb. Vasc. Biol..

[54] Naito R., Kasai T. (2015). Coronary artery disease in type 2 diabetes mellitus: Recent treatment strategies and future perspectives. World J. Cardiol..

[55] de Carvalho Cantarelli M.J., Castello H.J., Gonçalves R., Gioppato S., de Freitas Guimarães J.B., Ribeiro E.K.P., Vardi J.C.F., Maksud D., Navarro E.C. (2015). Preditores independentes de doença arterial coronária multiarterial: resultados do Registro Angiocardio. Rev. Bras. Cardiol. Invasiva.

[56] Marenzi G., Cabiati A., Assanelli E. (2012). Chronic kidney disease in acute coronary syndromes. World J. Nephrol..

[57] Saklayen M.G. (2018). The Global Epidemic of the Metabolic Syndrome. Current Hypertension Reports.

[58] Oikonomou E., Psaltopoulou T., Georgiopoulos G., Siasos G., Kokkou E., Antonopoulos A., Vogiatzi G., Tsalamandris S., Gennimata V., Papanikolaou A., Tousoulis D. (2018). Western Dietary Pattern Is Associated With Severe Coronary Artery Disease. Angiology.

[59] Collet J.P., Thiele H., Barbato E., Barthélémy O., Bauersachs J., Bhatt D.L., Dendale P., Dorobantu M., Edvardsen T., Folliguet T. (2021). 2020 ESC Guidelines for the management of acute coronary syndromes in patients presenting without persistent ST-segment elevation. Eur. Heart J..

[60] Chang H., Min J.K., Rao S.V., Patel M.R., Simonetti O.P., Ambrosio G., Raman S.V. (2012). Non-ST-segment elevation acute coronary syndromes targeted imaging to refine upstream risk stratification. Circ. Cardiovasc. Imaging.

[61] Li Z., Huang S., Yang R., Li J., Chen G. (2021). Long-term follow-up of diabetic patients with non-ST-segment elevation myocardial infarction. Am. J. Transl. Res..

[62] Balbi M.M., Scarparo P., Tovar M.N., Masdjedi K., Daemen J., Den Dekker W., Ligthart J., Witberg K., Cummins P., Wilschut J. (2022). Culprit Lesion Detection in Patients Presenting With Non-ST Elevation Acute Coronary Syndrome and Multivessel Disease. Cardiovasc. Revasc. Med..

[63] Baumann A.A.W., Mishra A., Worthley M.I., Nelson A.J., Psaltis P.J. (2020). Management of multivessel coronary artery disease in patients with non-ST-elevation myocardial infarction: a complex path to precision medicine. Ther. Adv. Chronic Dis..

[64] Zhang W., Mintz G.S., Cao Y., Matsumura M., Lee T., Hoshino M., Usui E., Kanaji Y., Murai T., Yonetsu T. (2022). Clinical determinants of coronary artery disease burden and vulnerability using optical coherence tomography co-registered with intravascular ultrasound. Coron. Artery Dis..

[65] Mintz G.S. (2015). Intravascular Imaging of Coronary Calcification and its Clinical Implications. JACC: Cardiovascular Imaging.

[66] Petousis S., Skalidis E., Zacharis E., Kochiadakis G., Hamilos M. (2023). The Role of Intracoronary Imaging for the Management of Calcified Lesions. J. Clin. Med..

[67] Wells G., Shea B., O’Connell D., Peterson J., Welch V., Losos M., Tugwell P. (2021). The Newcastle-Ottawa Scale (NOS) for assessing the quality of nonrandomised studies in meta-analyses. https://www.ohri.ca/programs/clinical_epidemiology/oxford.asp.

[68] McPheeters M.L., Sunil Kripalani M., Peterson N.B., Idowu R.T., Jerome R.N., Shannon Potter M.A., Jeffrey Andrews M.C. (2012). Closing the quality gap: revisiting the state of the science (vol. 3: quality improvement interventions to address health disparities). http://www.ahrq.gov.

[69] Higgins J., Thomas J., Chandler J., Cumpston M., Li T., Page M., Welch V. (2022).

[70] Duval S., Tweedie R. (2000). A Nonparametric “Trim and Fill” Method of Accounting for Publication Bias in Meta-Analysis. J. Am. Stat. Assoc..

