# Supplementary material for: Characteristics of calcified nodule attributable to culprit lesion in acute coronary syndrome: A systematic review and meta-analysis
Source: iScience. 2024 Jun 22;27(7):110351. doi: 10.1016/j.isci.2024.110351 (PMC11292520; doi:10.1016/j.isci.2024.110351)
Supplement: Document S1. Figures S1–S15 [file mmc1.pdf]

iScience, Volume *27*

Supplemental information

Characteristics of calcified nodule attributable to culprit lesion in acute coronary syndrome: A systematic review and meta-analysis

Roy Bagus Kurniawan, Pandit Bagus Tri Saputra, Alyaa Ulaa Dhiya Ul Haq, Dinda Dwi Purwati, Citrawati Dyah Kencono Wungu, Hendri Susilo, Mochamad Yusuf Alsagaff, Indah Mohd Amin, and Yudi Her Oktaviono

Supplementary Figures

Figure S1. Forest Plot of Leave-one-out Sensitivity Analysis Result of Pooled Prevalence of CN in ACS Patients, Related to Figure 2.

Figure S2. Forest Plot of Pooled Prevalence of CN in ACS Patients after Outliers' Exclusion, Related to Figure 2.

Figure S3. Funnel Plot of Pooled Prevalence of CN in ACS patients, Related to Figure 2.

Figure S4. Individual Forest Plot of Prevalence of CN in ACS Phenotypes and Culprit Arteries, Related to Figure 3. A.) In STEMI. B.) In NSTE-ACS. C.) In NSTE-MI. D.) In UA. E.) In LAD. F.) In LCx. G.) in RCA.

Figure S5. Individual Forest Plot of Prevalence of Obesity in CN (Top) and non-CN (Bottom) Group, Related to Figure 4.

Figure S6. Individual Forest Plot of Prevalence of Hypertension in CN (Top) and non-CN (Bottom) Group, Related to Figure 4.

Figure S7. Individual Forest Plot of Prevalence of Diabetes Mellitus in CN (Top) and non-CN (Bottom) Group, Related to Figure 4.

Figure S8. Individual Forest Plot of Prevalence of Dyslipidemia in CN (Top) and non-CN (Bottom) Group, Related to Figure 4.

Figure S9. Individual Forest Plot of Prevalence of History of MI in CN (Top) and non-CN (Bottom) Group, Related to Figure 4.

Figure S10. Individual Forest Plot of Prevalence of History of PCI in CN (Top) and non-CN (Bottom) Group, Related to Figure 4.

Figure S11. Individual Forest Plot of Prevalence of Multivessel Disease in CN (Top) and non-CN (Bottom) Group, Related to Figure 4.

Figure S12. Individual Forest Plot of Prevalence of Chronic Kidney Disease in CN (Top) and non-CN (Bottom) Group, Related to Figure 4.

Figure S13. Individual Forest Plot of Prevalence of Smoking in CN (Top) and non-CN (Bottom) Group, Related to Figure 4.

Figure S14. Funnel (top) and Forest Plot (bottom) of Trimmed and Filled Pooled Prevalence of CN in LCX, Related to Figure 3.

Figure S15. Funnel (top) and Forest Plot (bottom) of Trimmed and Filled Pooled Prevalence of CN in RCA, Related to Figure 3.

Figure S1. Forest Plot of Leave-one-out Sensitivity Analysis Result of Pooled Prevalence of CN in ACS Patients, Related to Figure 2.

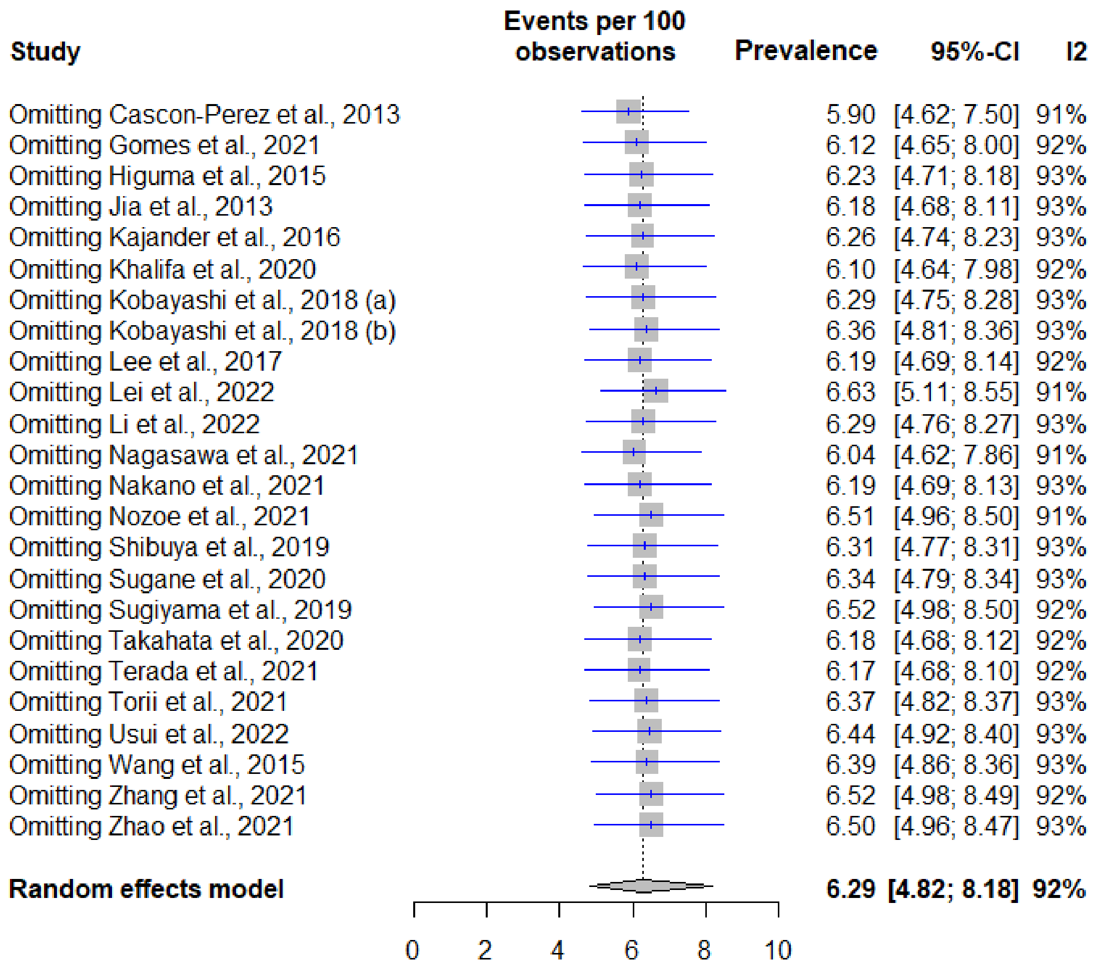

Figure S2. Forest Plot of Pooled Prevalence of CN in ACS Patients after Outliers' Exclusion, Related to Figure 2

Study	Events	Total	Prevalence	95%-CI	Weight
Cascon-Perez et al., 2013	23	98	23.47	[16.12; 32.85]	0.0%
Gomes et al., 2021	13	110	11.82	[6.99; 19.30]	5.7%
Higuma et al., 2015	9	111	8.11	[4.27; 14.85]	4.8%
Jia et al., 2013	10	104	9.62	[5.25; 16.96]	5.0%
Kajander et al., 2016	5	70	7.14	[3.00; 16.04]	3.2%
Khalifa et al., 2020	34	288	11.81	[8.56; 16.07]	0.0%
Kobayashi et al., 2018 (a)	21	333	6.31	[4.15; 9.48]	7.4%
Kobayashi et al., 2018 (b)	24	479	5.01	[3.38; 7.37]	7.8%
Lee et al., 2017	37	428	8.64	[6.33; 11.70]	8.8%
Lei et al., 2022	56	2593	2.16	[1.67; 2.80]	0.0%
Li et al., 2022	8	127	6.30	[3.18; 12.09]	4.5%
Nagasawa et al., 2021	61	436	13.99	[11.04; 17.57]	0.0%
Nakano et al., 2021	7	74	9.46	[4.58; 18.54]	4.0%
Nozoe et al., 2021	167	5332	3.13	[2.70; 3.63]	0.0%
Shibuya et al., 2019	28	483	5.80	[4.03; 8.27]	8.2%
Sugane et al., 2020	35	657	5.33	[3.85; 7.33]	8.8%
Sugiyama et al., 2019	36	1225	2.94	[2.13; 4.05]	0.0%
Takahata et al., 2020	27	297	9.09	[6.31; 12.93]	8.0%
Terada et al., 2021	15	156	9.62	[5.88; 15.34]	6.3%
Torii et al., 2021	25	524	4.77	[3.24; 6.97]	7.9%
Usui et al., 2022	3	110	2.73	[0.88; 8.11]	2.3%
Wang et al., 2015	2	62	3.23	[0.81; 12.00]	1.6%
Zhang et al., 2021	20	704	2.84	[1.84; 4.36]	0.0%
Zhao et al., 2021	12	408	2.94	[1.68; 5.11]	5.8%
**Random effects model**		**15209**	**6.58**	**[5.39; 8.01]**	**100.0%**
**Prediction interval**				**[3.61; 11.69]**	

Heterogeneity: $I^2 = 54\%$, $\tau^2 = 0.0781$, $p < 0.01$

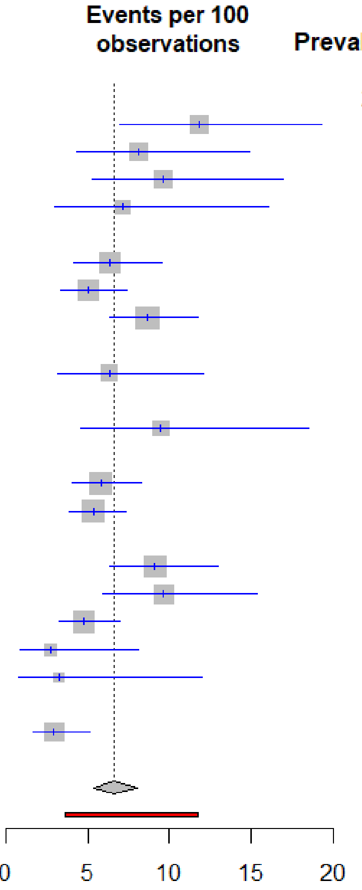

Figure S3. Funnel Plot of Pooled Prevalence of CN in ACS patients, Related to Figure 2

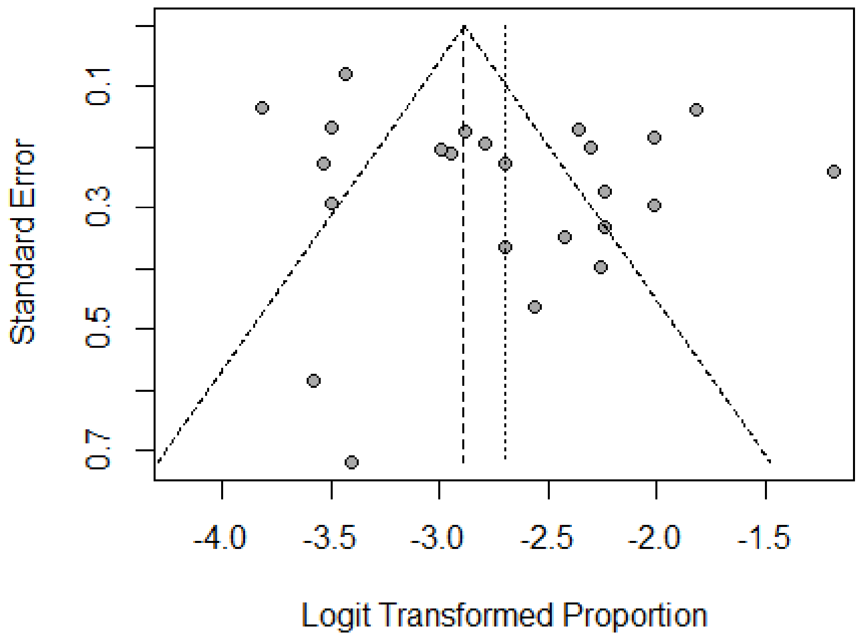

Figure S4. Individual Forest Plot of Prevalence of CN in ACS Phenotypes and Culprit Arteries, Related to Figure 3. A.) In STEMI. B.) In NSTE-ACS. C.) In NSTE-MI. D.) In UA. E.) In LAD. F.) In LCx. G.) in RCA.

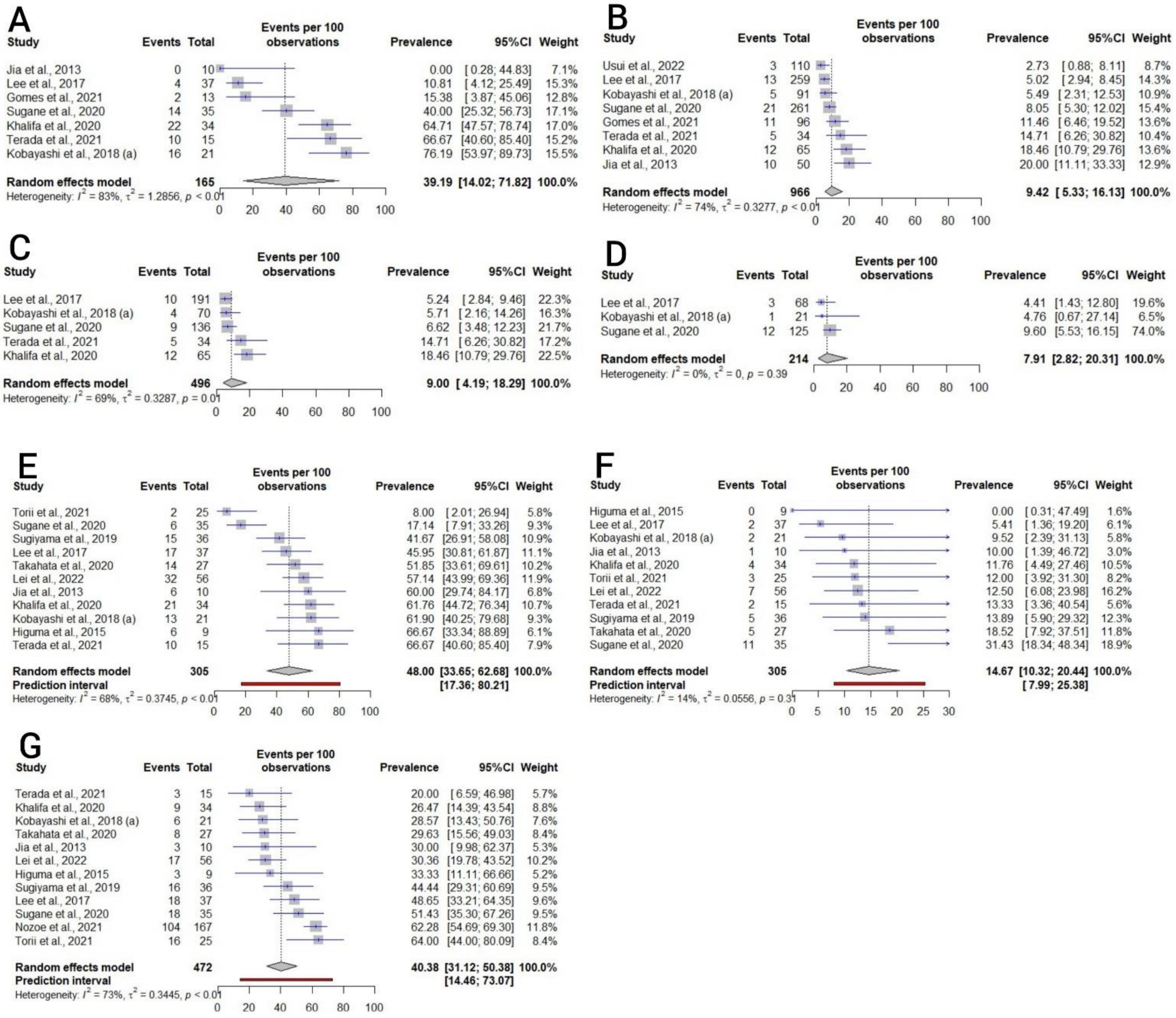

Figure S5. Individual Forest Plot of Prevalence of Obesity in CN (Top) and non-CN (Bottom) Group, Related to Figure 4

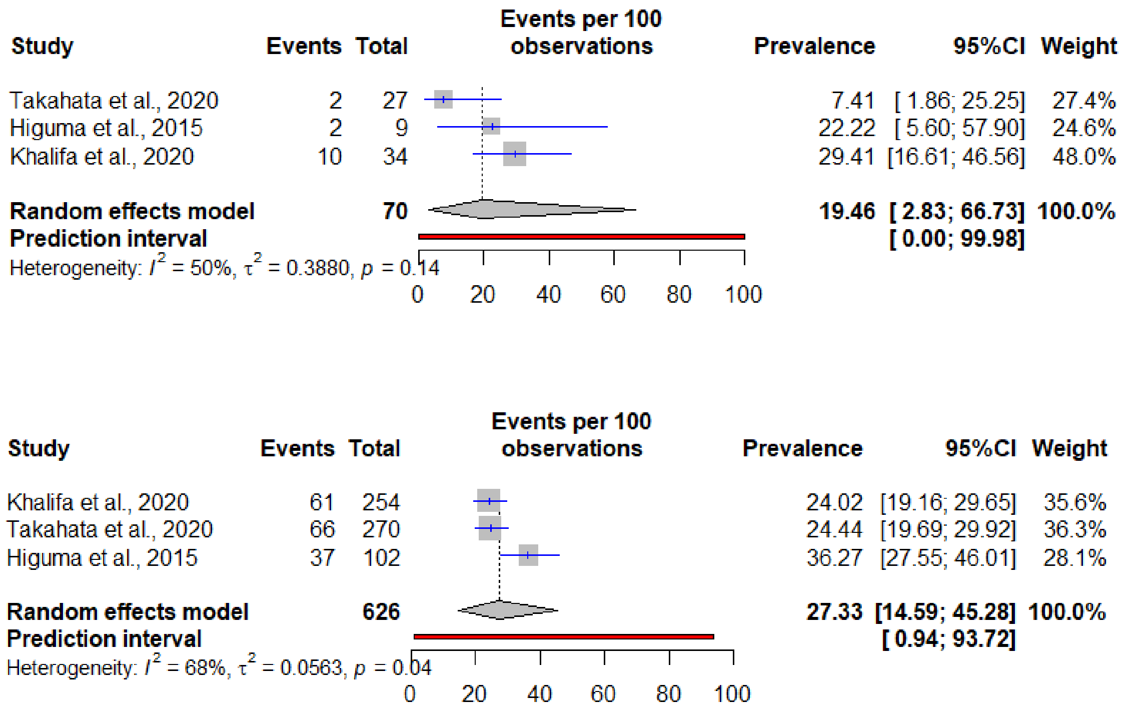

Figure S6. Individual Forest Plot of Prevalence of Hypertension in CN (Top) and non-CN (Bottom) Group, Related to Figure 4

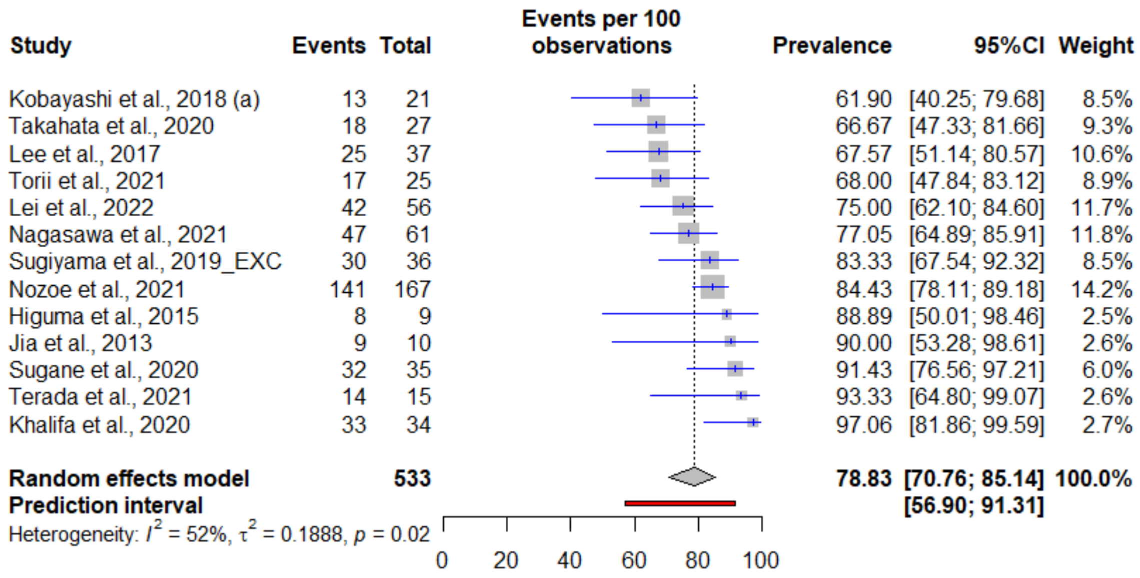

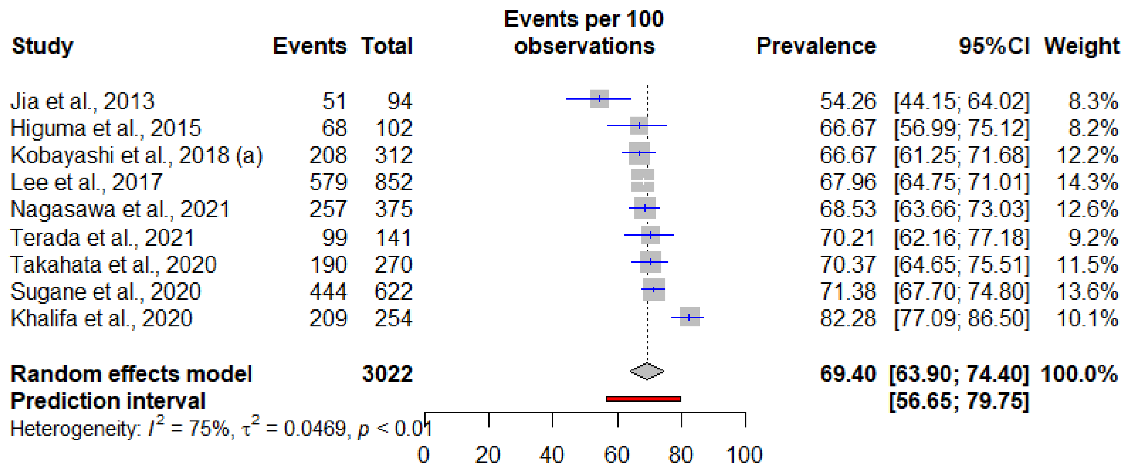

Figure S7. Individual Forest Plot of Prevalence of Diabetes Mellitus in CN (Top) and non-CN (Bottom) Group, Related to Figure 4

Study	Events	Total	Prevalence	95%CI	Weight
Lei et al., 2022	22	56	39.29	[27.46; 52.52]	10.3%
Jia et al., 2013	4	10	40.00	[15.83; 70.26]	1.8%
Takahata et al., 2020	11	27	40.74	[24.19; 59.70]	5.0%
Torii et al., 2021	11	25	44.00	[26.29; 63.38]	4.7%
Terada et al., 2021	7	15	46.67	[24.09; 70.70]	2.9%
Sugiyama et al., 2019_EXC	17	36	47.22	[31.74; 63.25]	6.9%
Sugane et al., 2020	17	35	48.57	[32.74; 64.70]	6.7%
Khalifa et al., 2020	17	34	50.00	[33.80; 66.20]	6.5%
Lee et al., 2017	19	37	51.35	[35.65; 66.79]	7.1%
Nozoe et al., 2021	89	167	53.29	[45.71; 60.73]	31.9%
Nagasawa et al., 2021	36	61	59.02	[46.37; 70.58]	11.3%
Higuma et al., 2015	6	9	66.67	[33.34; 88.89]	1.5%
Kobayashi et al., 2018 (a)	15	21	71.43	[49.24; 86.57]	3.3%
**Random effects model**		**533**	**50.76**	**[46.09; 55.42]**	**100.0%**
**Prediction interval**				**[45.95; 55.56]**	

Heterogeneity: $I^2 = 0\%$, $\tau^2 = 0$, $p = 0.48$

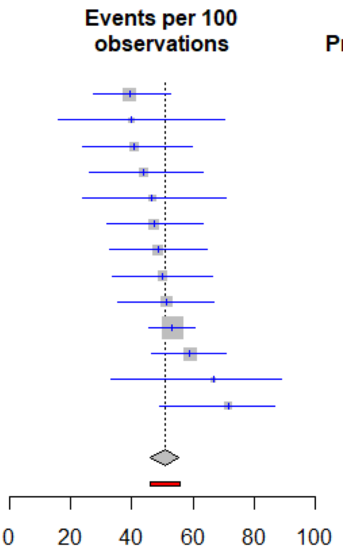

Study	Events	Total	Prevalence	95%CI	Weight
Jia et al., 2013	20	94	21.28	[14.16; 30.70]	3.9%
Terada et al., 2021	41	141	29.08	[22.18; 37.10]	6.4%
Lee et al., 2017	283	852	33.22	[30.13; 36.45]	19.3%
Sugane et al., 2020	216	622	34.73	[31.09; 38.56]	17.2%
Kobayashi et al., 2018 (a)	111	312	35.58	[30.46; 41.05]	12.1%
Takahata et al., 2020	99	270	36.67	[31.13; 42.58]	11.2%
Nagasawa et al., 2021	138	375	36.80	[32.07; 41.80]	13.6%
Khalifa et al., 2020	96	254	37.80	[32.04; 43.91]	10.8%
Higuma et al., 2015	40	102	39.22	[30.24; 48.98]	5.6%
**Random effects model**		**3022**	**34.62**	**[31.55; 37.83]**	**100.0%**
**Prediction interval**				**[28.99; 40.72]**	

Heterogeneity: $I^2 = 40\%$, $\tau^2 = 0.0093$, $p = 0.10$

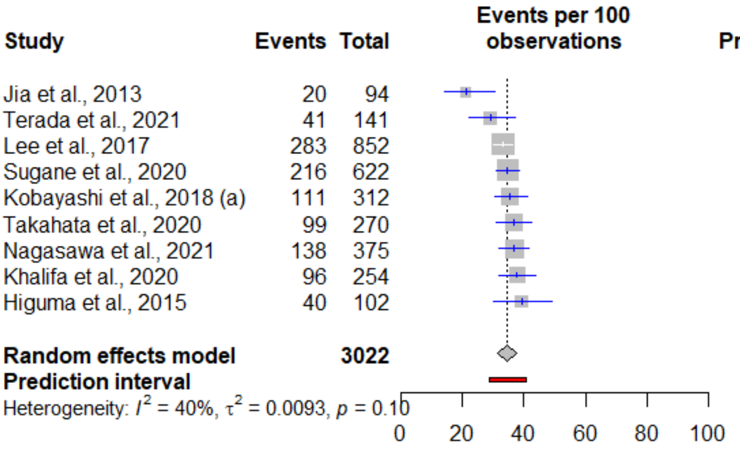

Figure S8. Individual Forest Plot of Prevalence of Dyslipidemia in CN (Top) and non-CN (Bottom) Group, Related to Figure 4

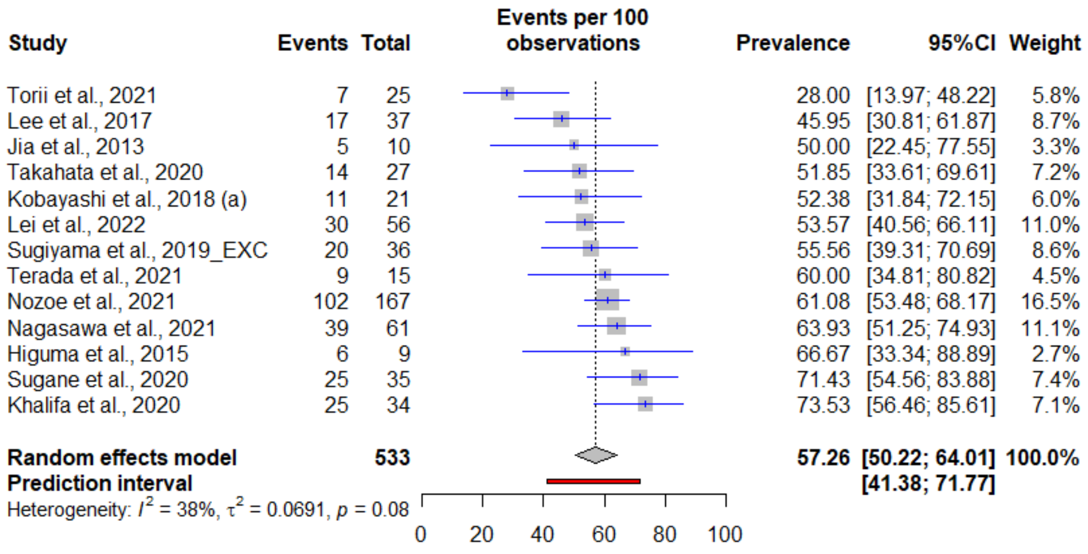

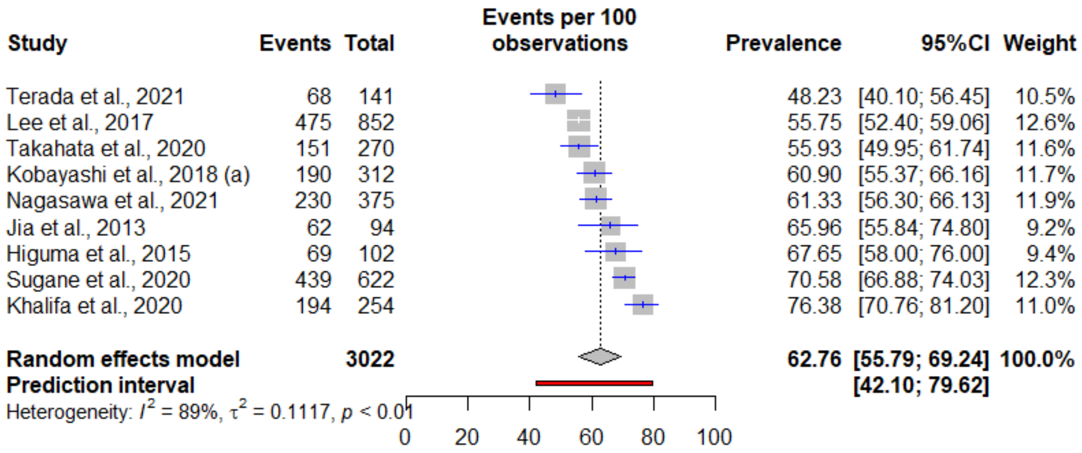

Figure S9. Individual Forest Plot of Prevalence of History of MI in CN (Top) and non-CN (Bottom) Group, Related to Figure 4

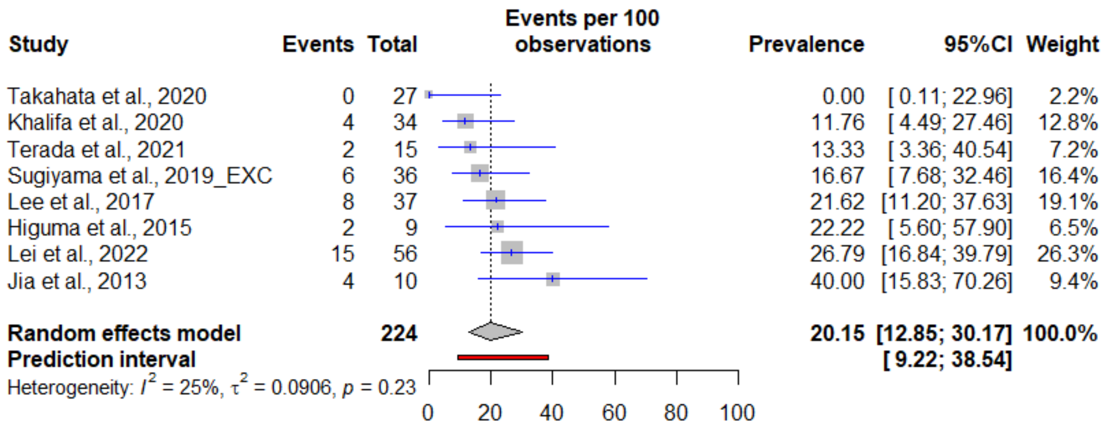

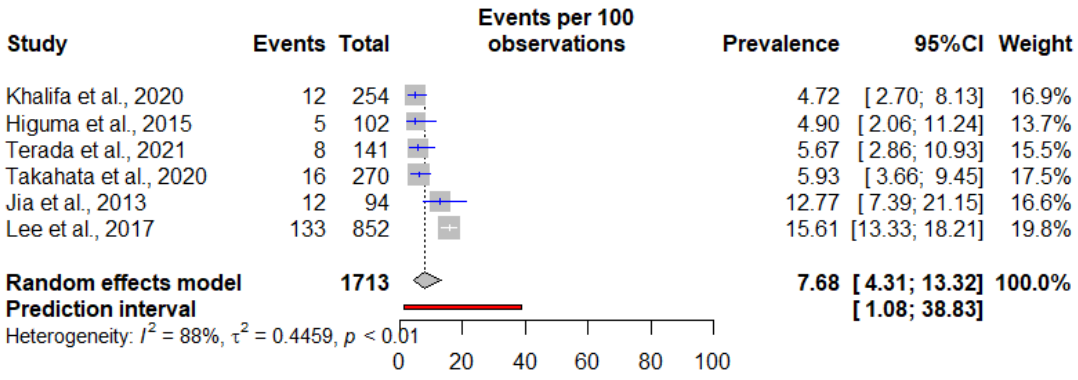

Figure S10. Individual Forest Plot of Prevalence of History of PCI in CN (Top) and non-CN (Bottom) Group, Related to Figure 4

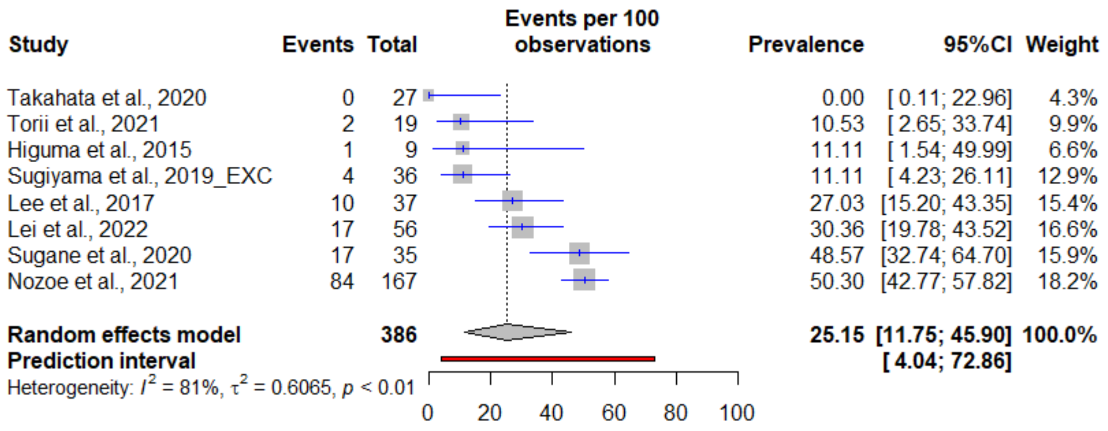

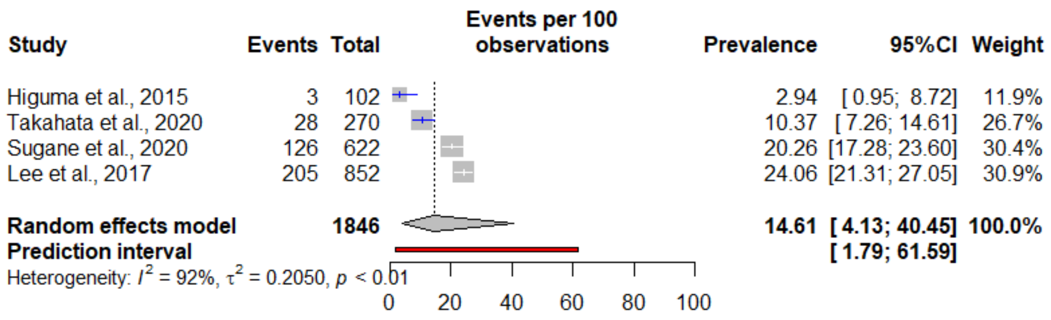

Figure S11. Individual Forest Plot of Prevalence of Multivessel Disease in CN (Top) and non-CN (Bottom) Group, Related to Figure 4

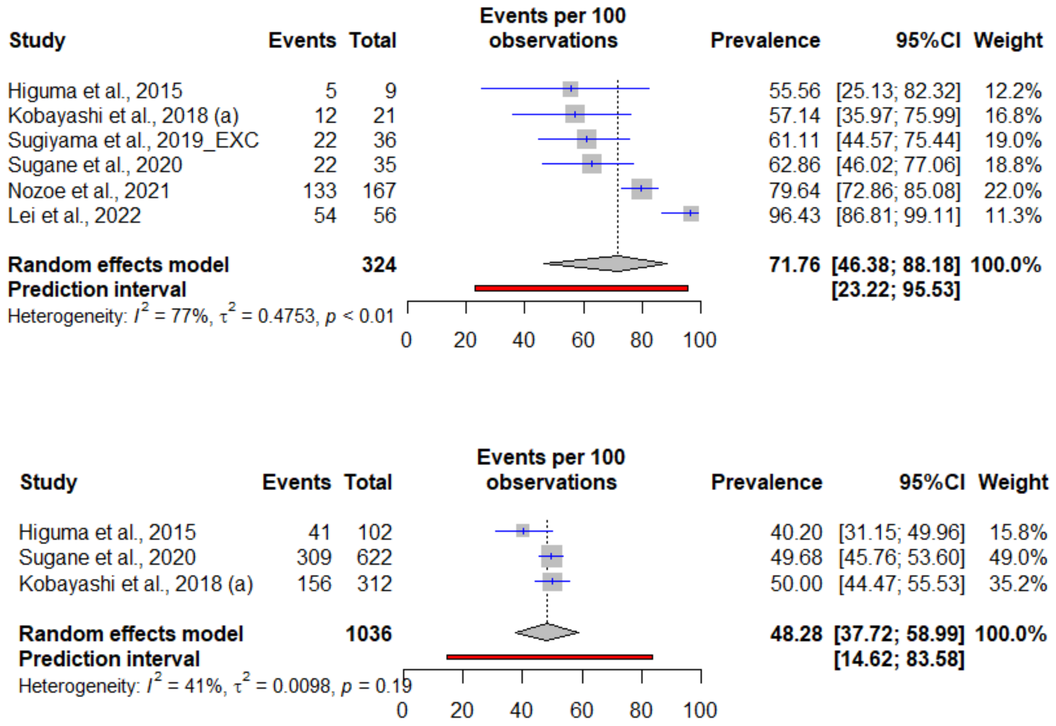

Figure S12. Individual Forest Plot of Prevalence of Chronic Kidney Disease in CN (Top) and non-CN (Bottom) Group, Related to Figure 4

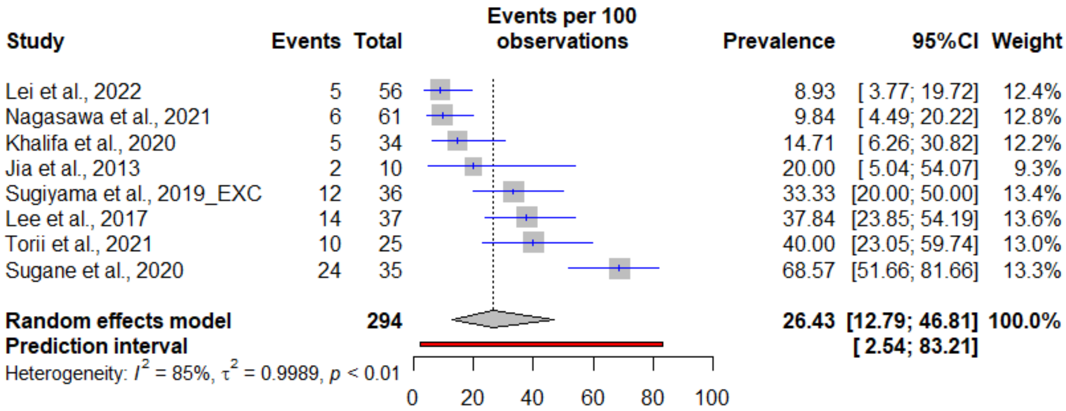

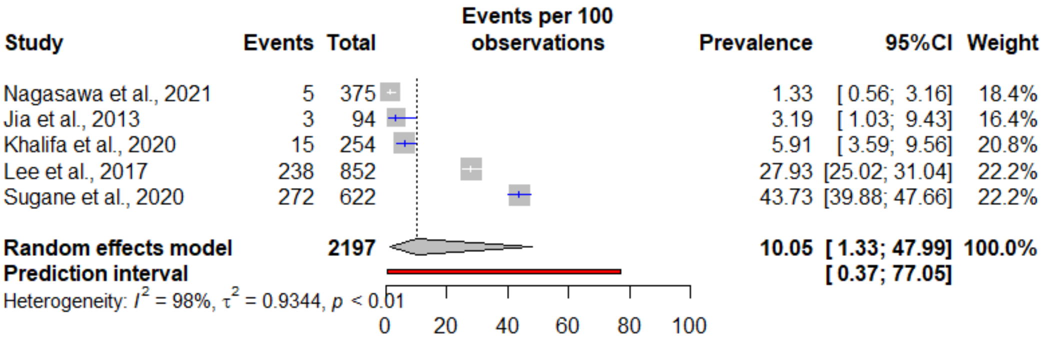

Figure S13. Individual Forest Plot of Prevalence of Smoking in CN (Top) and non-CN (Bottom) Group, Related to Figure 4

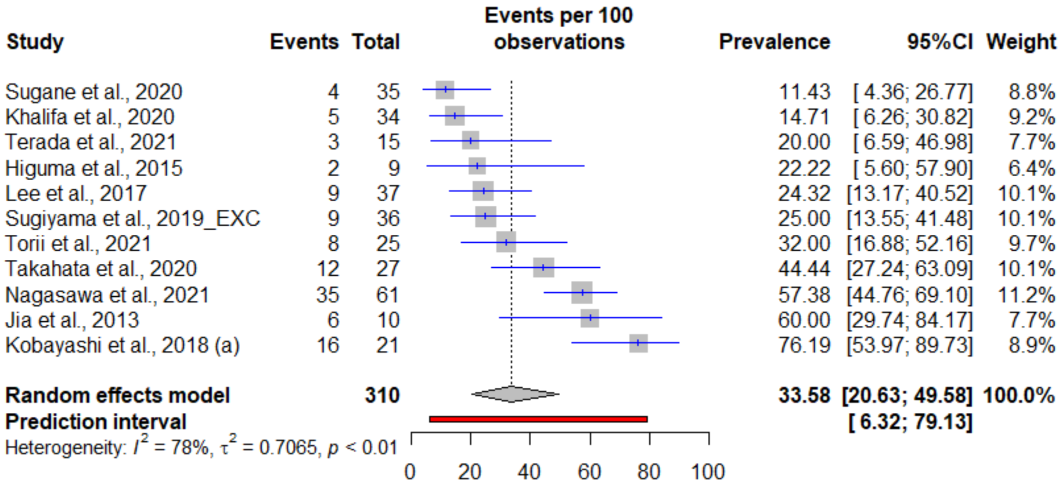

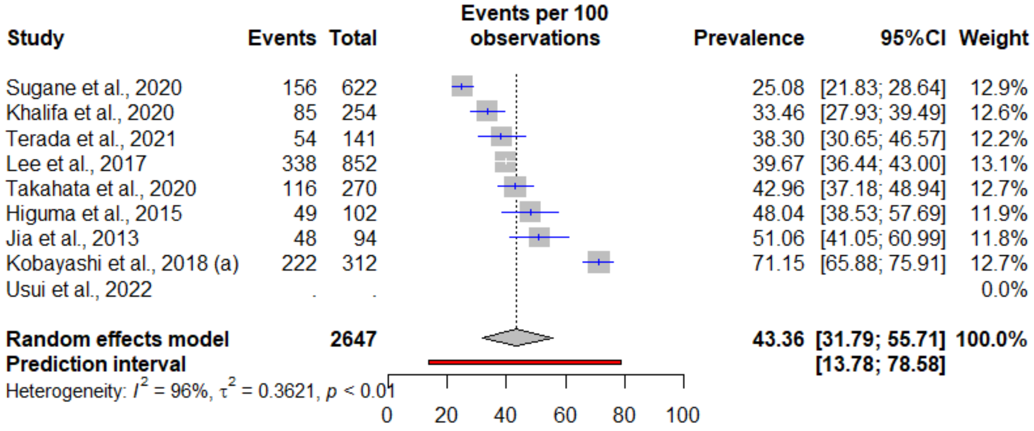

Figure S14. Funnel (top) and Forest Plot (bottom) of Trimmed and Filled Pooled Prevalence of CN in LCX, Related to Figure 3

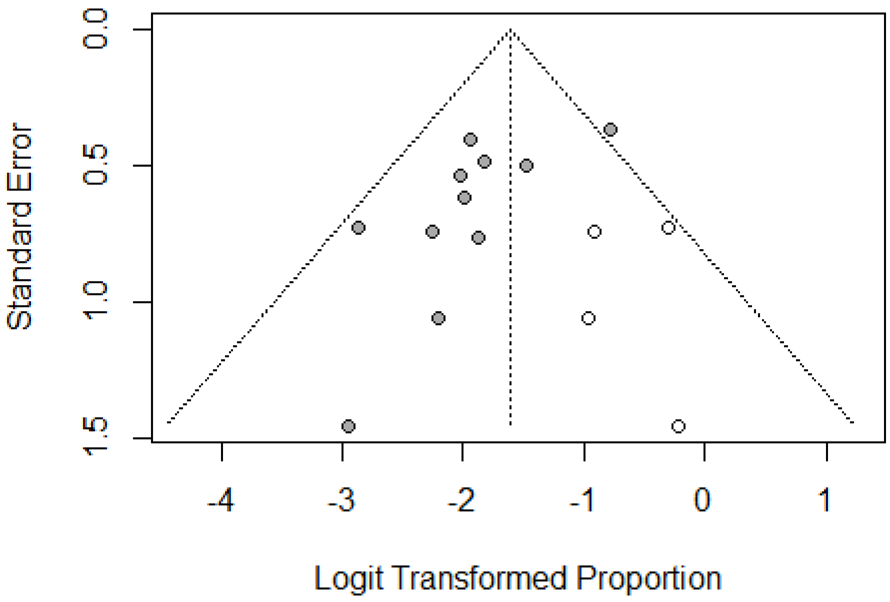

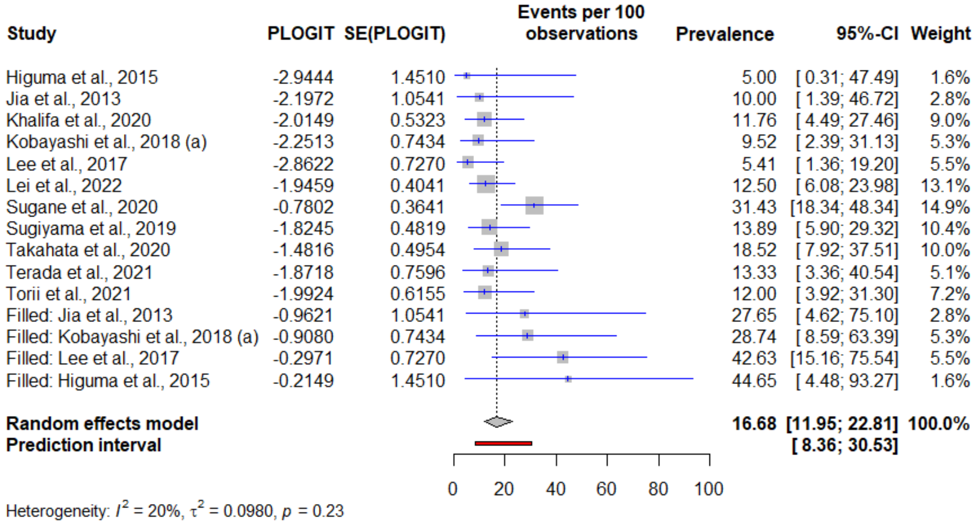

Figure S15. Funnel (top) and Forest Plot (bottom) of Trimmed and Filled Pooled Prevalence of CN in RCA, Related to Figure 3

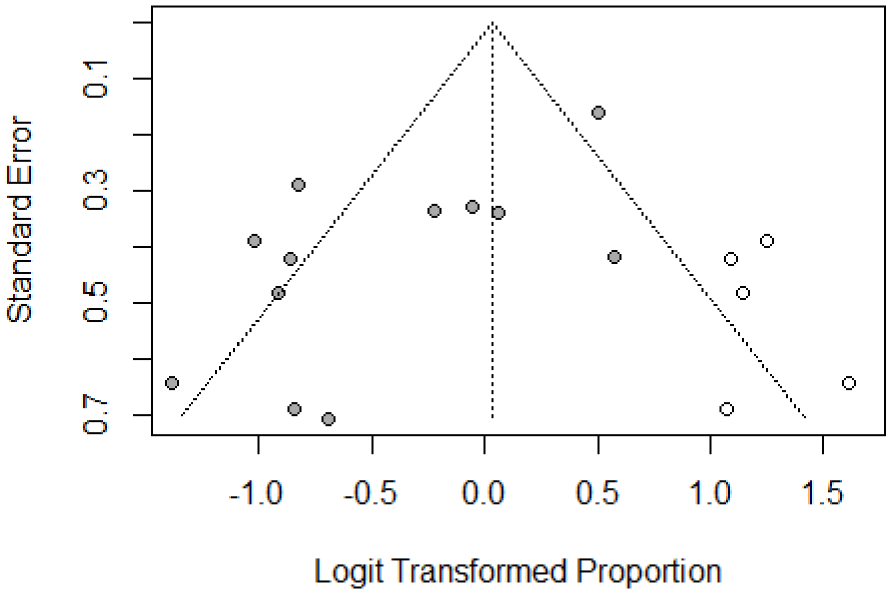

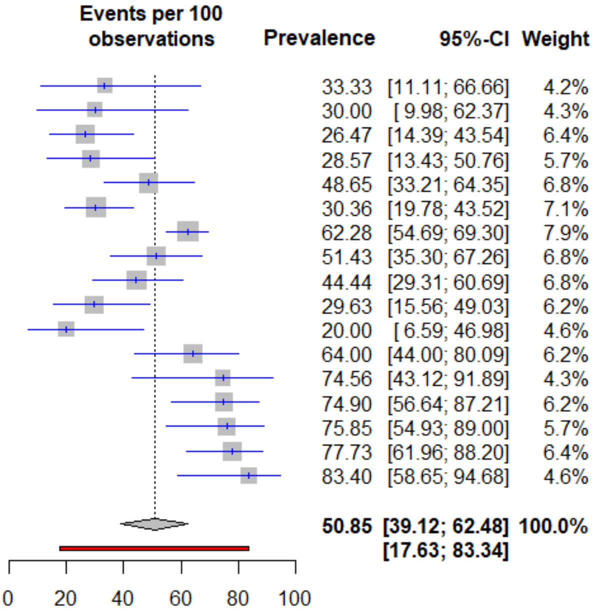

Study	PLOGIT	SE(PLOGIT)	Prevalence	95%-CI	Weight
Higuma et al., 2015	-0.6931	0.7071	33.33	[11.11; 66.66]	4.2%
Jia et al., 2013	-0.8473	0.6901	30.00	[9.98; 62.37]	4.3%
Khalifa et al., 2020	-1.0217	0.3887	26.47	[14.39; 43.54]	6.4%
Kobayashi et al., 2018 (a)	-0.9163	0.4830	28.57	[13.43; 50.76]	5.7%
Lee et al., 2017	-0.0541	0.3289	48.65	[33.21; 64.35]	6.8%
Lei et al., 2022	-0.8303	0.2906	30.36	[19.78; 43.52]	7.1%
Nozoe et al., 2021	0.5013	0.1597	62.28	[54.69; 69.30]	7.9%
Sugane et al., 2020	0.0572	0.3382	51.43	[35.30; 67.26]	6.8%
Sugiyama et al., 2019	-0.2231	0.3354	44.44	[29.31; 60.69]	6.8%
Takahata et al., 2020	-0.8650	0.4215	29.63	[15.56; 49.03]	6.2%
Terada et al., 2021	-1.3863	0.6455	20.00	[6.59; 46.98]	4.6%
Torii et al., 2021	0.5754	0.4167	64.00	[44.00; 80.09]	6.2%
Filled: Jia et al., 2013	1.0755	0.6901	74.56	[43.12; 91.89]	4.3%
Filled: Takahata et al., 2020	1.0932	0.4215	74.90	[56.64; 87.21]	6.2%
Filled: Kobayashi et al., 2018 (a)	1.1445	0.4830	75.85	[54.93; 89.00]	5.7%
Filled: Khalifa et al., 2020	1.2499	0.3887	77.73	[61.96; 88.20]	6.4%
Filled: Terada et al., 2021	1.6145	0.6455	83.40	[58.65; 94.68]	4.6%
**Random effects model**			**50.85**	**[39.12; 62.48]**	**100.0%**
**Prediction interval**				**[17.63; 83.34]**	

Heterogeneity: $I^2 = 78\%$, $\tau^2 = 0.5046$, $p < 0.01$
